# The role of sensory attenuation in symptomatic and healthy individuals: a scoping review

**DOI:** 10.3389/fnins.2025.1590127

**Published:** 2025-06-23

**Authors:** Luca Rossi, Francesco Cerritelli, Mick Thacker, Jorge E. Esteves

**Affiliations:** ^1^Clinical-Based Human Research Department, Foundation COME Collaboration, Pescara, Italy; ^2^College of Osteopathic Medicine, New York Institute of Technology, Old Westbury, NY, United States; ^3^Department of Physiotherapy, Royal College of Surgeons in Ireland, Dublin, Ireland; ^4^Escola Superior de Saúde Atlântica, Barcarena, Portugal

**Keywords:** active inference, manual therapy, predictive processing, sensory attenuation, chronic pain, musculoskeletal care, functional neurological disorders, persistent physical symptoms

## Abstract

**Background:**

Persistent physical symptoms (PPS), including functional neurological disorders (FND), chronic pain, and other neurological conditions [e.g., Parkinson’s disease (PD), Huntington’s disease (HD), autism spectrum disorder (ASD), and psychosis], present substantial challenges for healthcare systems due to their complex and multifaceted nature. These disorders often involve maladaptive sensory processing and heightened sensory perception, contributing to disability and psychological distress. Sensory attenuation (SA) is a neurophysiological mechanism that helps differentiate self-generated from external sensory stimuli, filtering irrelevant sensory input. Altered SA has been implicated in the pathophysiology of FND, chronic pain, and PPS, where impaired sensory modulation contributes to symptom persistence.

**Aims:**

This scoping review aimed to explore the role of SA in healthy individuals and those with FND, neurological disorders, and chronic pain. A secondary objective was to examine SA measurement techniques and their clinical relevance.

**Methods:**

Following the Joanna Briggs Institute (JBI) methodology and PRISMA-ScR guidelines, a comprehensive search of PubMed, ScienceDirect, and Google Scholar identified studies published between 2013 and 2023. Inclusion criteria encompassed investigations of SA in both symptomatic and healthy populations, focusing on FND, neurological disorders, and chronic pain. Data extraction highlighted SA mechanisms, assessment methods, and clinical implications for manual therapy and musculoskeletal (MSK) care.

**Results:**

A total of 62 studies involving 3,344 participants were included. Findings indicated that SA is essential for sensorimotor integration and the sense of agency in healthy individuals. However, disruptions in SA were consistently observed in FND, chronic pain, and neurological disorders, leading to sensory hypersensitivity, impaired motor control, and a distorted sense of agency. SA was assessed using methods such as the force-matching paradigm, electroencephalography (EEG), event-related potentials (ERPs), and functional magnetic resonance imaging (fMRI), providing insights into neurophysiological alterations.

**Conclusion:**

This review highlights the critical role of SA in adaptive sensory processing and its disruption in conditions like FND and chronic pain. Integrating SA-based interventions, such as sensorimotor retraining and affective touch, into manual therapy and MSK care may help recalibrate sensory processing and improve patient outcomes. Future research should focus on standardizing SA assessments and exploring its modulation in clinical settings to enhance person-centered therapeutic approaches.

## 1 Introduction

Persistent physical symptoms (PPS), also known as persistent somatic symptoms, are distressing complaints—such as chronic pain, fatigue, or dizziness—that last for at least 6 months and often lack a clear medical explanation (Kube et al., 2020; Löwe et al., 2024; Van den Bergh et al., 2017). They may develop after infections, injuries, or stressful events, or arise without an obvious trigger. PPS are closely linked to significant disability and healthcare burden, and as symptoms persist, their connection to identifiable pathology tends to weaken, making diagnosis and treatment more difficult (Henningsen et al., 2018; Löwe et al., 2024). These symptoms overlap with functional neurological disorder (FND), as both conditions involve disabling physical experiences without structural pathology and share common underlying mechanisms, including dysfunctions in brain networks and psychological contributors (Gilmour et al., 2020).

FND presents with genuine neurological symptoms—such as seizures, movement abnormalities, or sensory disruptions—in the absence of structural brain damage. It is now understood as a disorder of brain function, with diagnosis based on identifiable clinical features rather than exclusion of other conditions (Gilmour et al., 2020; Mishra and Pandey, 2022). Research shows that FND is as prevalent in neurological clinics, with 5–15% of patients requiring assistance (Finkelstein et al., 2025), as multiple sclerosis and involves disruptions in attention, brain connectivity and predictive processing.

PPS, which include conditions like chronic fatigue syndrome, fibromyalgia, and irritable bowel syndrome, often co-occur with or resemble FND. These disorders can range in severity from mild to debilitating, significantly affecting patients’ lives and representing a large portion of healthcare referrals (Chalder et al., 2023; Swainston et al., 2022). The chronic nature and complexity of these conditions are associated with disability, unemployment, and psychological distress, reinforcing the deep interconnection between mental and physical health (Broddadóttir et al., 2021; den Boer et al., 2022). Managing PPS and FND requires comprehensive, multidisciplinary care strategies to address their multifactorial nature (Husain and Chalder, 2021; Kube et al., 2020; Rask et al., 2023).

The relationship between PPS and FND is shaped by psychological factors such as stress, trauma, and emotional strain, which can intensify symptoms and hinder recovery (Löwe et al., 2024). These influences may lead to the development of maladaptive coping strategies and maintain illness behaviours. Some individuals may transition from PPS to FND under sustained psychological and functional strain, while others may experience FND symptoms without any identifiable pathology. This complexity highlights the importance of a biopsychosocial approach to both diagnosis and treatment. FND, as a spectrum disorder, is influenced by chronic pain, anxiety, depression, and diagnostic uncertainty (Hallett et al., 2022). Given the burden of these conditions—especially among women—early, targeted interventions addressing neurobiological, psychological, and social domains are urgently needed (Milano et al., 2023; Palmer et al., 2023).

Effective treatment for PPS and FND often involves a multidisciplinary approach, integrating cognitive-behavioural therapy, physical therapy, and patient education to enhance coping strategies, symptom management, and overall functioning (Löwe et al., 2024). Importantly, patients’ beliefs and expectations play a pivotal role in symptom perception and management, underscoring the necessity of open communication and collaboration between patients and healthcare providers to ensure effective treatment (Löwe et al., 2024).

Emerging models, such as predictive processing and active inference, offer valuable insights into understanding the mechanisms underlying PPS and FND by conceptualising the brain as a hierarchical, multilevel predictive machine. Predictive processing posits that the brain continuously generates models to anticipate sensory inputs and minimise prediction errors by integrating feedback loops between different neural layers of the cortex (Bubić et al., 2009). This process involves the interplay of top-down predictions from higher brain areas and bottom-up sensory information to refine these predictions, resulting in an adaptive and dynamic internal model (Teufel and Fletcher, 2020; Friston, 2009). At lower levels, the brain predicts interoceptive (internal bodily), proprioceptive (body position and movement), and exteroceptive (external sensory) inputs. When discrepancies arise between predicted and actual sensory inputs, prediction-error signals are generated, prompting adjustments to the generative model to improve future predictions (Clark, 2013; Friston, 2005; Smith et al., 2019). Importantly, the ability for prediction errors to update the generative models is dependent on precision-weighting; briefly, only those errors that have low-variance (high precision) produce alterations in neural circuitry and function (see for full description Haarsma et al., 2018). This process enables continuous appropriate learning and adaptation, where perceptual inference (updating the internal model) and active inference (acting on the environment to align it with predictions) minimise prediction errors and optimise behaviour (Esteves et al., 2022; Venter, 2021).

Active inference (AInf) extends this framework by explaining how organisms adapt their behaviour and beliefs to reduce discrepancies between internal models and the external world, thereby integrating processes such as perception, action selection, attention, and emotion regulation (Parr et al., 2022). AInf is grounded in the principle of minimising variational free energy through Bayesian inference, where organisms modify their beliefs (perceptual adaptation) or alter the environment (behavioural adaptation) to reduce uncertainty (Parr et al., 2022). This process is guided by a generative model that predicts sensory inputs and physiological states, enabling both reactive and proactive regulation of behaviour and internal states. The concept of expected free energy plays a crucial role in evaluating potential outcomes of different actions, while the Markov blanket offers a degree of autonomy by separating internal and external states, allowing for greater self-regulation (Parr et al., 2022). Additionally, AInf extends to interoceptive and emotional regulation, elucidating how the nervous system and associated systems predict and manages physiological conditions and their associated emotional responses, which is vital for maintaining homeostasis and adaptive functioning. Through AInf, Bayesian information flow can be mapped onto cortical neurobiological computations through constant-time (predictive coding) or discrete-time (Markov decision processes) formulations. In this context, the dopaminergic (ventral tegmental area/substantia nigra pars compacta), cholinergic (Nucleus basalis) and noradrenergic (locus coeruleus) neuromodulator systems have gained importance. Due to their location in the brainstem, they influence the accuracy of sensory signals, action/control activities and model predictions with respect to the surrounding environment. In particular, the dopaminergic system has been associated with both the genesis of movement and the selectivity of action. Instead, the cholinergic system would appear to be implicated in top-down mechanisms of cortico-sensory regulation and attention selectivity. Finally, the noradrenergic system would seem to encode the accuracy of the model’s predictions with respect to the variability of the environment, allowing the model to learn and update itself (Limanowski et al., 2024).

Within this conceptual framework, sensory attenuation emerges as a crucial mechanism for modulating sensory information and maintaining an accurate sense of self and agency (Esteves et al., 2022). Sensory attenuation refers to the nervous systems ability to selectively filter and downregulate the intensity of internally generated sensory signals, distinguishing them from externally generated stimuli. This process ensures that self-generated actions, such as movements or thoughts, do not overwhelm the sensory system (Harrison et al., 2021; Parthasharathy et al., 2022). However, when sensory attenuation fails, as seen in conditions like FND and chronic pain, patients experience an exaggerated awareness of bodily sensations, contributing to symptom persistence and discomfort (Esteves et al., 2022; Palmer et al., 2023).

For example, individuals with FND often exhibit impaired sensory attenuation, resulting in difficulties differentiating self-generated movements from external stimuli, which may contribute to their symptoms (Harrison et al., 2021). Similarly, in chronic pain conditions, the breakdown of sensory attenuation leads to an amplified perception of nociceptive signals, perpetuating hypervigilance and discomfort (Esteves et al., 2022). Understanding sensory attenuation, therefore, offers a pathway to deciphering how predictive processes become disrupted in these conditions, making it a critical target for therapeutic intervention.

Clinicians, particularly manual therapists, can leverage principles of predictive processing and active inference to modify patients’ generative models and recalibrate sensory attenuation. This involves engaging patients in person-centred and dyadic therapeutic relationships that facilitate recalibration of sensory processing, using techniques such as affective touch, guided movement, and verbal communication to help patients re-learn how to filter and modulate sensory input effectively via the AInf processes outlined previously (Esteves et al., 2022). By guiding patients through this process, clinicians can help restore the body’s ability to “disappear” into the background of conscious experience, thereby reducing symptom intensity and improving functional outcomes (Vasil et al., 2020).

Digital health interventions, particularly virtual reality (VR), present a promising modality for recalibrating sensory attenuation in individuals with PPS and chronic musculoskeletal pain. VR creates immersive, multisensory environments that provide congruent proprioceptive, visual, and auditory stimuli—ideal for guiding predictive coding processes by stabilising prior expectations and minimising prediction errors (Pretat et al., 2025). For example, virtual embodiment training protocols have shown efficacy in reducing pain perception and enhancing motor engagement by simulating body-related experiences under controlled, low-threat conditions. These interventions align with predictive processing models where altered interoception and disrupted sensory attenuation contribute to pain chronification (Darnall et al., 2020). Furthermore, recent reviews and pilot studies highlight how VR can support long-term sensorimotor retraining and attentional redirection without the physical constraints typical in conventional rehabilitation (Saby et al., 2024). By allowing therapeutic exposure to graded activities, VR not only enhances accessibility and efficiency in clinical care, but also empowers patient self-management in ecologically valid settings. These capabilities make VR an increasingly relevant tool for both clinicians and researchers exploring the neural mechanisms of pain and sensory integration (Guerra-Armas et al., 2023).

Despite the growing understanding of sensory attenuation’s role in these conditions, there is a significant gap in the literature regarding its manifestation in symptomatic versus healthy individuals, particularly within the context of musculoskeletal and person-centred care. This scoping review aims to address this gap by exploring the role of sensory attenuation in healthy individuals, FND, other neurological disorders (e.g., Parkinson’s disease (PD), Huntington’s disease (HD), autism spectrum disorder (ASD), and psychosis), and chronic pain. Additionally, it seeks to investigate how sensory attenuation is measured in both healthy and symptomatic subjects, assessing the implications and applicability of these tests.

By examining sensory attenuation through the lens of predictive processing and active inference, this review aims to inform clinical practice, enhance patient-practitioner communication, and lay the groundwork for future research in musculoskeletal care. Ultimately, this exploration will contribute to the development of innovative therapeutic strategies grounded in predictive processing models, supporting a more holistic and person-centred approach to managing complex conditions like PPS and FND.

## 2 Methods

This scoping review was conducted following the Joanna Briggs Institute (JBI) methodology for scoping reviews (Peters et al., 2020) and is reported in accordance with the Preferred Reporting Items for Systematic Reviews and Meta-Analyses extension for scoping reviews (PRISMA-ScR) checklist (Tricco et al., 2018).

### 2.1 Protocol and registration

A protocol for this scoping review was developed prior to the commencement of the study, adhering to the JBI guidelines. The protocol was registered under the number https://doi.org/10.17605/OSF.IO/TR5Z8.

### 2.2 Review questions

The primary review question is: what is the role of sensory attenuation in healthy individuals, PPS, FND, neurological disorders, and/or chronic pain?

The secondary review question is: how is sensory attenuation measured in healthy and symptomatic individuals, and what are the implications and applicability of these tests?

### 2.3 Eligibility criteria

The inclusion criteria were established using the JBI’s population, concept, and context (PCC) framework (Peters et al., 2015).

*Population*: Paediatric, adult, and elderly individuals of any gender, who are healthy or have FND, non-functional neurological disorders, and/or chronic pain. Studies analysing these populations through the lens of osteopathy and enactivism were included.

*Concept*: Studies that explore (1) the role of sensory attenuation in healthy and symptomatic individuals; (2) methods of assessing sensory attenuation phenomena; and (3) the implications and applicability of clinical tests for sensory attenuation in patients.

*Context*: Studies conducted within a neuroscientific framework, including those related to manual and non-manual clinical medicine.

#### 2.3.1 Types of sources

This review considered all types of studies, including: experimental and interventional studies [randomised controlled trials (RCTs), N-of-1 trials], observational studies (case reports, case series, cross-sectional, cohort, and case–control studies), systematic reviews, scoping reviews, and narrative reviews, qualitative studies, grey literature that meets the inclusion criteria. Only studies published in English between 2013 and 2023 were included to ensure the most current evidence.

#### 2.3.2 Exclusion criteria

Studies were excluded if they were: not published in English, published before 2013, conducted on animals, *in vitro*, or on robots, pre-prints not certified by peer review and undergraduate or doctoral dissertations and did not analyse the variable of interest (sensory attenuation).

### 2.4 Information sources

A comprehensive literature search was conducted across the following electronic databases: PubMed, ScienceDirect and Google Scholar.

Additional sources included grey literature and contacting authors to request full-text articles for two publications related to sensory attenuation that were not open access.

### 2.5 Search strategy and selection process

The research methodology for this scoping review consisted of the following steps:

#### 2.5.1 Databases searched and search filters

The databases used for the screening of the English-language scientific literature were PubMed, ScienceDirect, Semantic Scholar, and Google Scholar, with a time span from 2013 to 2023. No registries or websites were utilised.

#### 2.5.2 Keywords and search strings

The search strings were structured by combining “Sensory attenuation” with each of the other keywords using the Boolean operator AND. Table 1 shows the specific search strings used.

**Table 1:** Literature search strategy used for the current review.

Search strings (Sensory attenuation + Boolean operator + keyword):
“Sensory attenuation” AND “Persistent physical symptoms”
“Sensory attenuation” AND “Pain”
“Sensory attenuation” AND “Chronic pain”
“Sensory attenuation” AND “Predictive processing”
“Sensory attenuation” AND “Predictive coding”
“Sensory attenuation” AND “Quantitative sensory testing”
“Sensory attenuation” AND “Active inference”
“Sensory attenuation” AND “Free energy principle”
“Sensory attenuation” AND “Generative models”
“Sensory attenuation” AND “Osteopathy”
“Sensory attenuation” AND “Enactivism”
“Sensory attenuation” AND “Manual therapy”
“Sensory attenuation” AND “Visual domain”
“Sensory attenuation” AND “Auditory domain”
“Sensory attenuation” AND “Touch domain”
“Sensory attenuation” AND “Generative model”

#### 2.5.3 Selection process

During the research process, reports (electronic or paper documents) identified and corresponding to the selection criteria of the scoping review were saved and catalogued by an individual reviewer using Zotero reference management software. To be saved in Zotero, the records (titles and abstracts) of the reports indexed in these databases had to contain all keywords of the search string in at least one of them.

After the article selection process, two independent reviewers examined the studies. Any exclusions of scientific articles (e.g., duplicates, studies not meeting the inclusion criteria) were recorded and reported in the scoping review.

#### 2.5.4 Data management

**Figure 1:**
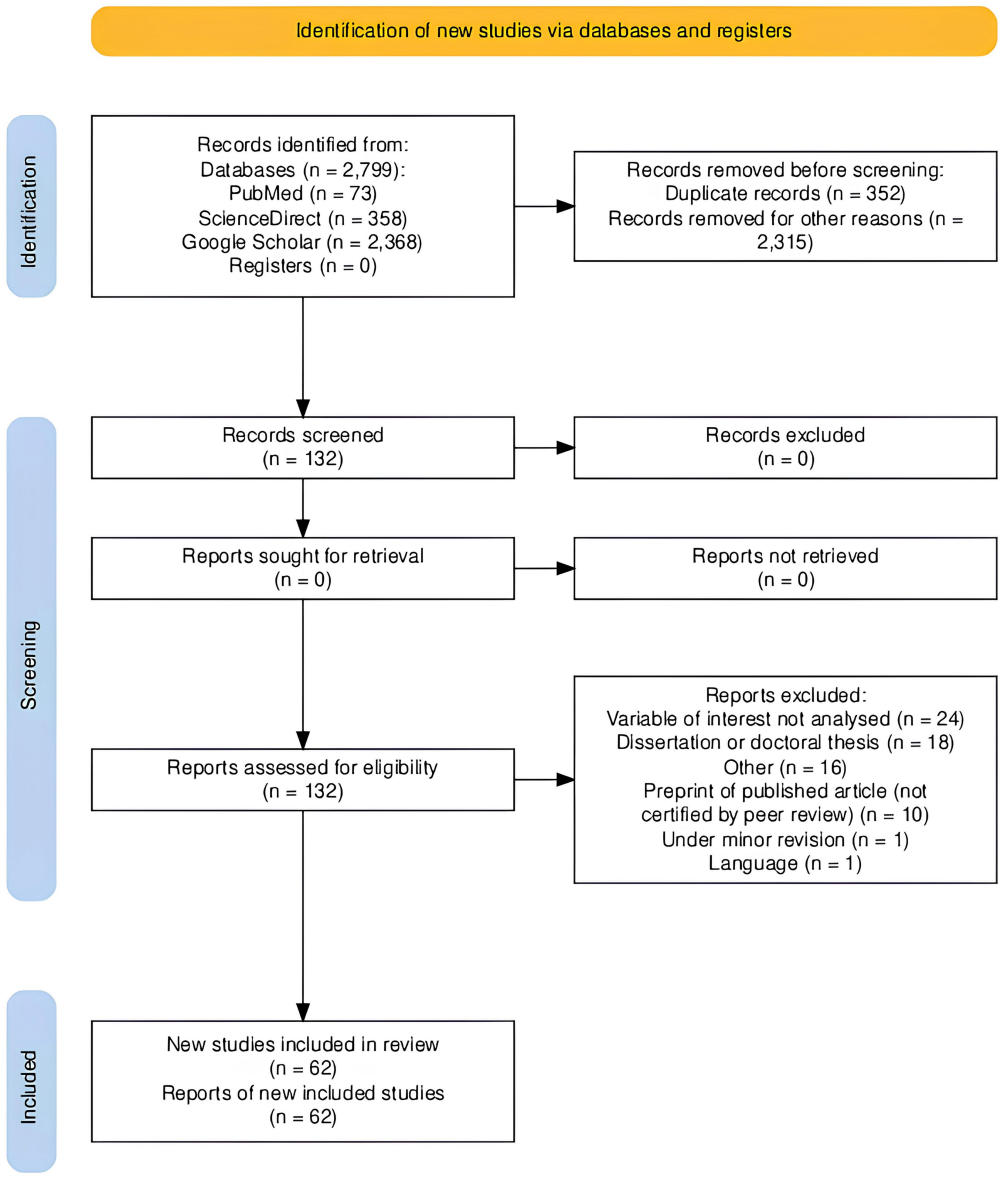
PRISMA-ScR flow diagram.

The identification process of the articles considered eligible for the study was systematically and sequentially reported. This process was presented within a flowchart following the Preferred Reporting Items for Systematic Reviews and Meta-Analyses extension for scoping reviews (PRISMA-ScR) guidelines (see Figure 1).

### 2.6 Selection of sources of evidence

All identified citations were collated, and duplicates were removed. Titles and abstracts were screened independently by two reviewers against the inclusion criteria. Potentially relevant studies were retrieved in full text and assessed in detail against the inclusion criteria. Reasons for exclusion of sources at the full-text screening stage were recorded and reported in the scoping review. Any disagreements between the reviewers (LR and JE) were resolved through discussion or consultation with a third reviewer (FC).

## 3 Results

### 3.1 Selection of sources of evidence

The literature search across PubMed, ScienceDirect, and Google Scholar identified a total of 2,799 records. After removing 352 duplicates, 2,447 records remained. Applying the inclusion and exclusion criteria resulted in the removal of 2,315 records.

A total of 132 full-text articles were assessed for eligibility. Seventy articles were excluded for the following reasons: variable of interest not analysed (*n* = 24), dissertations or doctoral theses (*n* = 18), other reasons (n = 16), preprints not certified by peer review (*n* = 10), under minor revision (*n* = 1), and language not in English (*n* = 1).

Ultimately, 62 articles met the inclusion criteria and were included in the scoping review. These articles were analysed to address the primary review question: “What is the role of sensory attenuation in healthy individuals, FND, neurological disorders, and/or chronic pain?” and the secondary question: “How is sensory attenuation measured in healthy and symptomatic individuals, and what are the implications and applicability of these tests?”

The process of article selection is summarised in the PRISMA-ScR flow diagram (Figure 1).

### 3.2 Characteristics of included studies

The 62 included studies encompassed a total of 3,344 participants, comprising 1,372 men, 1,738 women, two non-binary individual, one participant who identified as neither man nor woman, and 231 participants whose gender was not specified, with a mean age of 27 years. Ten studies were excluded from demographic calculations due to their design: six literature reviews (Hughes et al., 2013b; Boehme and Olausson, 2022; Brown et al., 2013; Kearney and Brittain, 2021; Ciaunica et al., 2022; Adams et al., 2013), one mini-review (Kiepe et al., 2021), one observational study (Porciuncula et al., 2020), one experimental study without demographic data (Gentsch et al., 2015), and one hypothesis and theory article (Windt et al., 2014).

#### 3.2.1 Study designs

The included studies comprised various designs: Experimental studies (*n* = 51), including two within-subject designs (Burin et al., 2017; Lubinus et al., 2022) and one randomised block design (Orepic et al., 2021); Reviews (*n* = 6) (Hughes et al., 2013b; Boehme and Olausson, 2022; Brown et al., 2013; Kearney and Brittain, 2021; Ciaunica et al., 2022; Adams et al., 2013); Mini-review (*n* = 1) (Kiepe et al., 2021); Observational study (*n* = 1) (Porciuncula et al., 2020); Cross-sectional study (*n* = 1) (McNaughton et al., 2022a); Proof-of-concept study (*n* = 1) (Fritsch et al., 2021); Hypothesis and theory article (*n* = 1) (Windt et al., 2014).

#### 3.2.2 Geographical distribution

**Figure 2:**
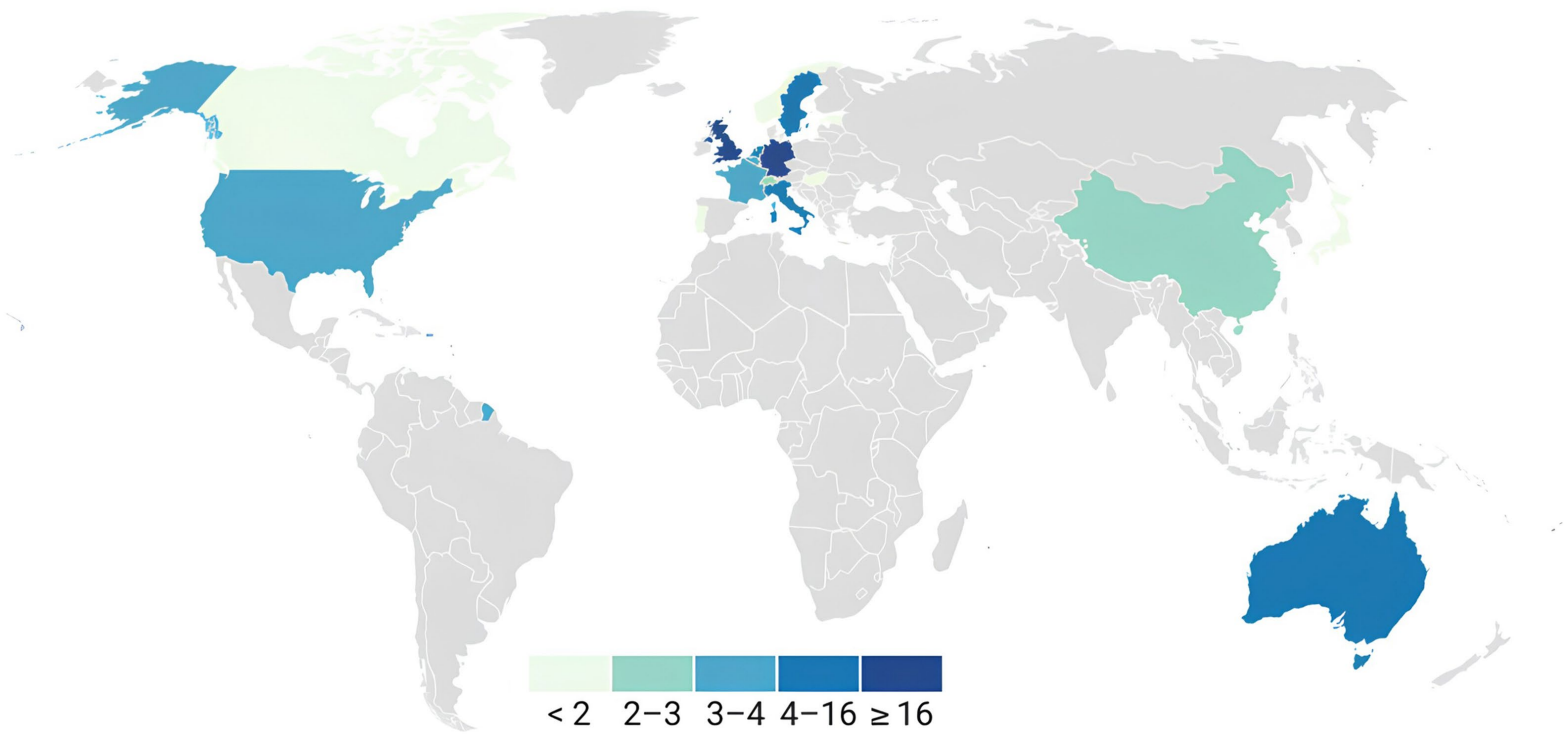
Geographical distribution of the included papers. The data of the first author of each article were used to represent the geographical distribution. United Kingdom (17): Palmer et al. (2016), Macerollo et al. (2015), Brown et al. (2013), Kearney and Brittain (2021), Wolpe et al. (2018), Hua et al. (2020), Hughes (2015), Ciaunica et al. (2022), Finnemann et al. (2021), Stenner et al. (2014a), Stenner et al. (2014b), Cao et al. (2017), Hua et al. (2023), Adams et al. (2013), Gentsch et al. (2015), Pareés et al. (2014), and Cao and Gross (2015); Germany (16): Storch and Zimmermann (2022), Knoetsch and Zimmermann (2021), Stenner et al. (2014a), Stenner et al. (2014b), Lubinus et al. (2022), Abbasi and Gross (2019), Fritz et al. (2022), Fritsch et al. (2021), Fritz and Zimmermann (2023), Kiepe et al. (2021), Windt et al. (2014), Weller et al. (2017), Jo et al. (2019), Kiepe et al. (2023), Klaffehn et al. (2019), and Schwarz et al. (2018); Australia (7): McNaughton et al. (2022a), Harrison et al. (2021), Han et al. (2021), McNaughton et al. (2021), McNaughton et al. (2022b), Mifsud et al. (2018), and Mifsud and Whitford (2017); Italy (4): Macerollo et al. (2015), Burin et al. (2017), Pyasik et al. (2021), and Parthasharathy et al. (2022); Sweden (4): Lalouni et al. (2021), Kilteni and Ehrsson (2017), Boehme et al. (2019), and Boehme and Olausson (2022); Netherlands (4): Richter and de Lange (2019), van Laarhoven et al. (2019), Dogge et al. (2019), and van Elk et al. (2014); USA (3): Bolt and Loehr (2021), Lee and Schmit (2018), and Porciuncula et al. (2020); Belgium (3): Pinto et al. (2021), Vasser et al. (2019), and Parthasharathy et al. (2022); France (3): Roussel et al. (2014), Hughes et al. (2013a), and Hughes et al. (2013b); China (2): Hua et al. (2023) and Dong and Bao (2021); Switzerland (2): van Elk et al. (2014) and Orepic et al. (2021); Canada (1): Loehr (2013); Estonia (1): Vasser et al. (2019); Japan (1): Nuruki et al. (2019); Norway (1): Csifcsák et al. (2018); Portugal (1): Ciaunica et al. (2022); Hungary (1): Csifcsák et al. (2018). Articles whose authors reported a link to more than one geographical area were included in the overall count. The studies in question are as follows: UK—Italy: Macerollo et al. (2015); UK— Germany: Stenner et al. (2014b); UK—Portugal: Ciaunica et al. (2022); UK—China: Hua et al. (2023); UK—Germany: Stenner et al. (2014a); UK—Belgium: Parthasharathy et al. (2022); Belgium—Estonia: Vasser et al. (2019); Norway—Hungary: Csifcsák et al. (2018).

The studies were conducted across various countries (Figure 2).

#### 3.2.3 Participant characteristics

**Figure 3:**
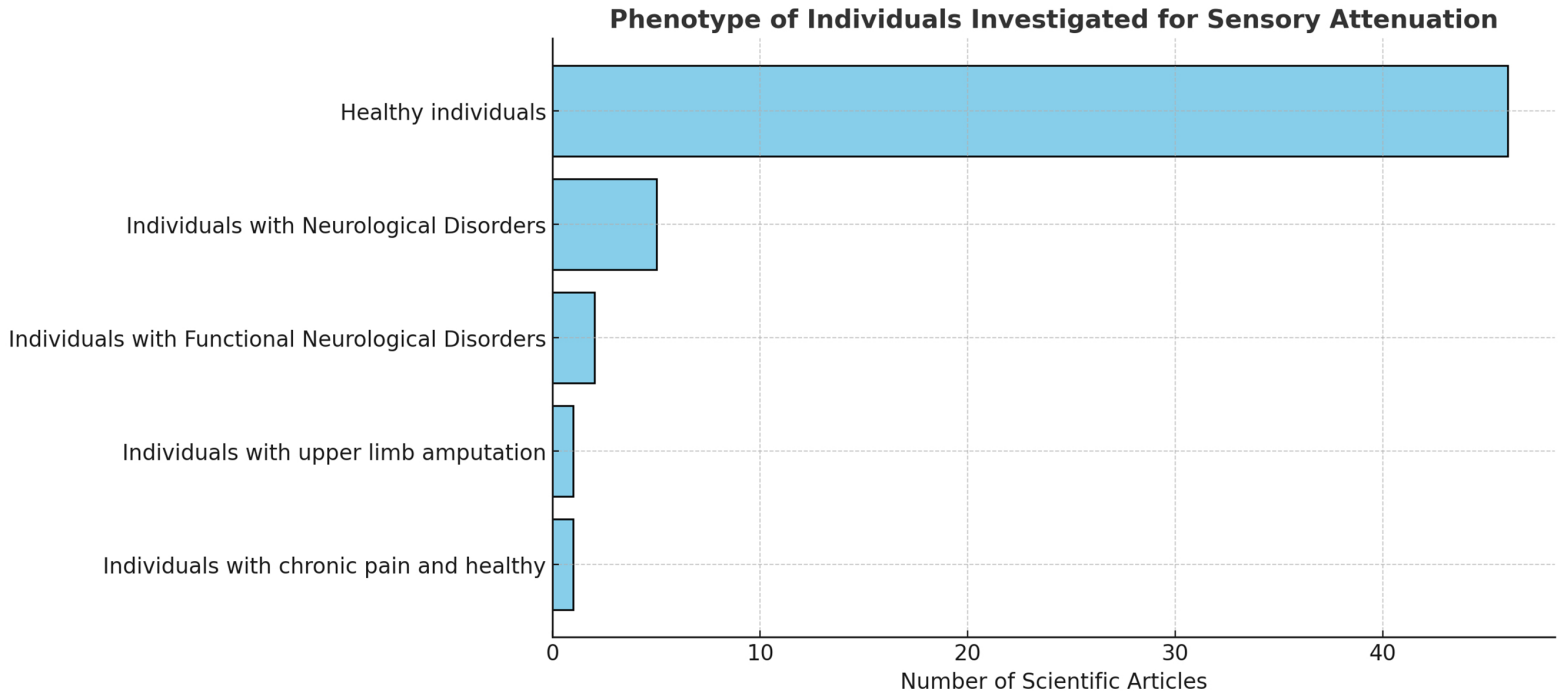
Clustered bar chart.

Most studies recruited healthy participants (*n* = 46). Of these, seven studies included participants with normal or corrected-to-normal vision (Orepic et al., 2021; Fritz et al., 2022; Lubinus et al., 2022; Storch and Zimmermann, 2022; Schwarz et al., 2018; Vasser et al., 2019; Cao and Gross, 2015). One study analysed individuals with upper limb amputation (Fritsch et al., 2021), and one study compared patients with chronic pain to healthy controls (McNaughton et al., 2022a). Ten articles examined sensory attenuation through the lens of FND and neurological impairments (see Figure 3).

### 3.3 Synthesis of results

**Figure 4:**
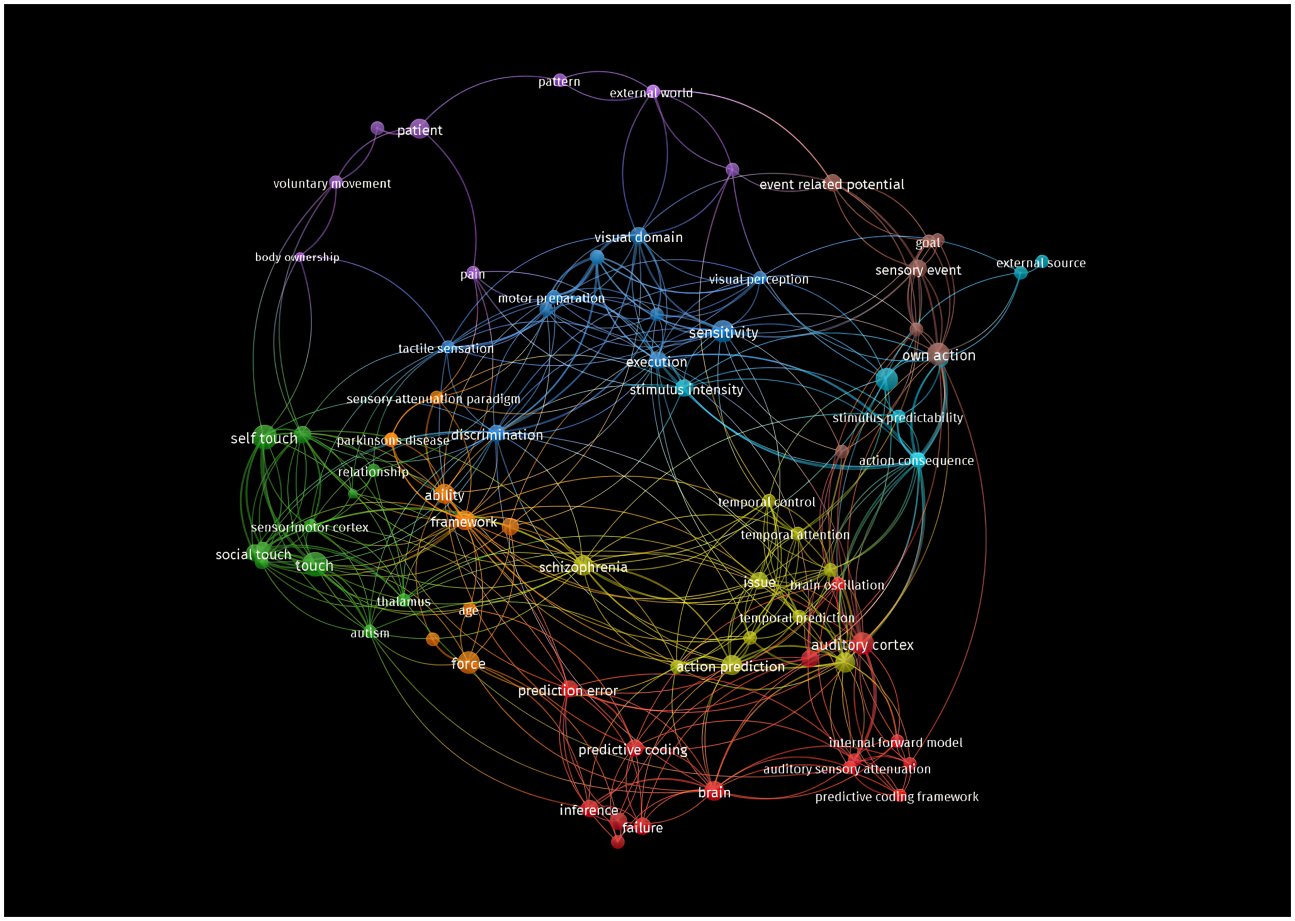
Network visualisation. The label and circle size of an element are determined by the weight of the element. Specifically, the weight of the article is directly proportional to the size of the label and the circle of the item. The proximity of one element to another and the thickness of the lines connecting them are indicators of a strong correlation.

Figure 4 and Supplementary material provides a visual representation of the clinical areas and phenomena investigated in the studies included in this review. Other data visualisations are available in the Supplementary material.

#### 3.3.1 Sensory attenuation in healthy individuals

##### 3.3.1.1 Visual domain

Ten studies investigated sensory attenuation (SA) in the visual domain among healthy individuals. These studies utilised methods such as virtual reality (VR), saccade paradigms, and discrimination tasks of visual stimuli (Dong and Bao, 2021; Vasser et al., 2019; Kiepe et al., 2023; Schwarz et al., 2018; Storch and Zimmermann, 2022; Csifcsák et al., 2018; Richter and de Lange, 2019; Mifsud et al., 2018; Roussel et al., 2014; Lubinus et al., 2022).

Research findings in this domain are varied. Some studies suggest that SA occurs in the visual domain and is influenced by internal predictive signals, such as proprioception and attention (Storch and Zimmermann, 2022; Kiepe et al., 2023). For instance, Kiepe et al. (2023) found that the ability to discriminate stimulus intensity is modulated by internal predictive cues, potentially associated with proprioceptive attention. Storch and Zimmermann (2022) demonstrated that temporal attention, guided by temporal predictability, can modulate the strength of SA.

Conversely, other studies indicate that SA does not automatically occur for all foreseeable consequences of a voluntary action in the visual domain (Schwarz et al., 2018). The variability in findings suggests that SA in the visual domain is complex and may depend on factors such as task requirements, stimulus predictability, and the temporal relationship between action and perception (Csifcsák et al., 2018; Richter and de Lange, 2019).

##### 3.3.1.2 Auditory domain

Nineteen studies explored SA in the auditory domain. These studies examined neural responses to self-generated versus externally generated sounds, often using electroencephalography (EEG) to measure event-related potentials (ERPs) such as the N1 and P2 components (Cao et al., 2017; Abbasi and Gross, 2019; Stenner et al., 2014a; Mifsud and Whitford, 2017; Klaffehn et al., 2019; van Elk et al., 2014; Harrison et al., 2021; Hughes et al., 2013a; Dogge et al., 2019; Han et al., 2021; Orepic et al., 2021; Weller et al., 2017; Stenner et al., 2014b; Cao and Gross, 2015; Loehr, 2013; Bolt and Loehr, 2021).

Findings generally indicate that SA manifests as attenuation of neural responses to self-generated sounds compared to externally generated sounds. This attenuation is thought to result from predictive mechanisms in the brain that anticipate the sensory consequences of one’s own actions (Cao et al., 2017; Abbasi and Gross, 2019). For example, Abbasi and Gross (2019) highlighted the role of beta oscillations in regulating top-down interactions between motor and auditory cortices, supporting the predictive coding framework.

Some studies explored SA in joint action contexts. Loehr (2013) and Bolt and Loehr (2021) investigated whether SA occurs when individuals jointly produce sounds with others. They found that SA can help individuals distinguish between self-generated and partner-generated sounds during coordinated activities.

##### 3.3.1.3 Tactile-proprioceptive domain

Seventeen studies investigated SA in the tactile-proprioceptive domain, using methods such as force-matching paradigms, gentle touch, and nerve stimulation (Kilteni and Ehrsson, 2017; Palmer et al., 2016; Lee and Schmit, 2018; Parthasharathy et al., 2022; Knoetsch and Zimmermann, 2021; Lalouni et al., 2021; Pinto et al., 2021; Windt et al., 2014; Hughes, 2015; Burin et al., 2017; Nuruki et al., 2019; McNaughton et al., 2021; Fritz and Zimmermann, 2023; Gentsch et al., 2015; Boehme et al., 2019; Pyasik et al., 2021; Fritsch et al., 2021).

Results suggest that SA leads to reduced perception of self-generated touch compared to externally generated touch. This phenomenon is attributed to internal predictive models that anticipate sensory consequences of voluntary actions, leading to attenuation of expected sensations (Kilteni and Ehrsson, 2017; Palmer et al., 2016). SA in this domain is spatially specific (Knoetsch and Zimmermann, 2021) and can be influenced by factors such as age (Parthasharathy et al., 2022) and the presence of pain (Lalouni et al., 2021).

For example, Parthasharathy et al. (2022) found that older adults exhibited higher levels of SA compared to younger adults, potentially due to age-related proprioceptive deficits. Lalouni et al. (2021) demonstrated that SA occurs for self-generated pressure pain conditions, which could have clinical implications for pain management.

#### 3.3.2 Sensory attenuation in functional neurological disorders and neurological disorders

Ten studies examined SA in populations with FND and other neurological conditions, including PD, HD, ASD, and individuals at high clinical risk for psychosis (Macerollo et al., 2015; Pareés et al., 2014; Wolpe et al., 2018; Kearney and Brittain, 2021; Porciuncula et al., 2020; Hua et al., 2023; Adams et al., 2013; Ciaunica et al., 2022; Finnemann et al., 2021; van Laarhoven et al., 2019).

##### 3.3.2.1 Functional neurological disorders

In studies focusing on FND, patients exhibited reduced SA compared to healthy controls. Pareés et al. (2014) found that patients with FND did not overestimate the force to be used in a force-matching task, indicating a significant loss of SA. This reduction in SA may be related to an altered sense of agency, suggesting why individuals with FND perceive abnormal movements as unintentional.

Macerollo et al. (2015) corroborated these findings, showing that patients with functional movement disorders exhibited decreased SA, potentially linked to impaired motor control and agency.

##### 3.3.2.2 Parkinson’s disease

In PD, Wolpe et al. (2018) investigated the effects of dopaminergic treatment on SA. They found that medication increased SA and improved the accuracy of sensory and motor predictions. This suggests that bradykinesia in PD may be associated with impaired sensory and motor anticipation, which can be modulated by dopamine.

Kearney and Brittain (2021) discussed the role of SA in PD, highlighting that patients may have difficulty integrating sensory stimuli, which affects movement production and perception.

##### 3.3.2.3 Autism spectrum disorder

Studies on ASD revealed mixed results. Finnemann et al. (2021) found that individuals with ASD showed intact predictive and postdictive mechanisms of SA, suggesting that a general deficit in predictive processing is unlikely. However, van Laarhoven et al. (2019) reported that individuals with ASD exhibited impaired SA for self-generated sounds, indicating deficits in motor-auditory prediction and supporting the notion of atypical sensory processing in ASD.

##### 3.3.2.4 High clinical risk for psychosis

Hua et al. (2023) examined SA deficits in individuals at high clinical risk for psychosis and those with a first episode of psychosis. They found alterations in SA-related brain activity in auditory and thalamic regions, suggesting that early stages of psychosis are characterised by impaired ability to predict sensory consequences of actions.

##### 3.3.2.5 Sensory attenuation in chronic pain

McNaughton et al. (2022a) compared SA between individuals with chronic pain and healthy controls using a force-matching task. While no significant differences were found in the magnitude of SA between groups, patients with chronic pain exhibited greater variability in SA, indicating altered sensory processing and potential disruptions in predictive mechanisms.

#### 3.3.3 Measurement of sensory attenuation

A more granular understanding of the measurement techniques for sensory attenuation is essential for translational application in clinical contexts. The force-matching paradigm remains the most widely used behavioural method, offering simplicity, low cost, and ecological validity. It enables quantification of sensory prediction errors by comparing externally versus self-generated force perception (Hughes et al., 2013b). However, its reliance on voluntary motor output limits use in patients with motor deficits or paediatric/geriatric populations. In contrast, neurophysiological methods such as EEG and magnetoencephalography (MEG) provide time-sensitive indices of SA via ERPs or frequency band modulations (Sakkalis, 2011). These modalities are highly sensitive to millisecond-level cortical dynamics, making them ideal for capturing prediction error signalling (Geukes et al., 2013; Sharon et al., 2007). Nonetheless, they require specialised equipment and expertise, and are less portable. Functional magnetic resonance imaging (fMRI), while offering excellent spatial resolution, is limited by low temporal sensitivity, high cost, and constrained ecological validity (Warbrick, 2022). Therefore, while EEG and force-matching offer potential for clinical adaptation, particularly with mobile EEG and tablet-based interfaces, fMRI and MEG remain best suited for mechanistic and research purposes. Future research should prioritise the development of portable, multimodal assessments that combine behavioural and electrophysiological indices to track SA in diverse patient populations.

**Figure 5:**
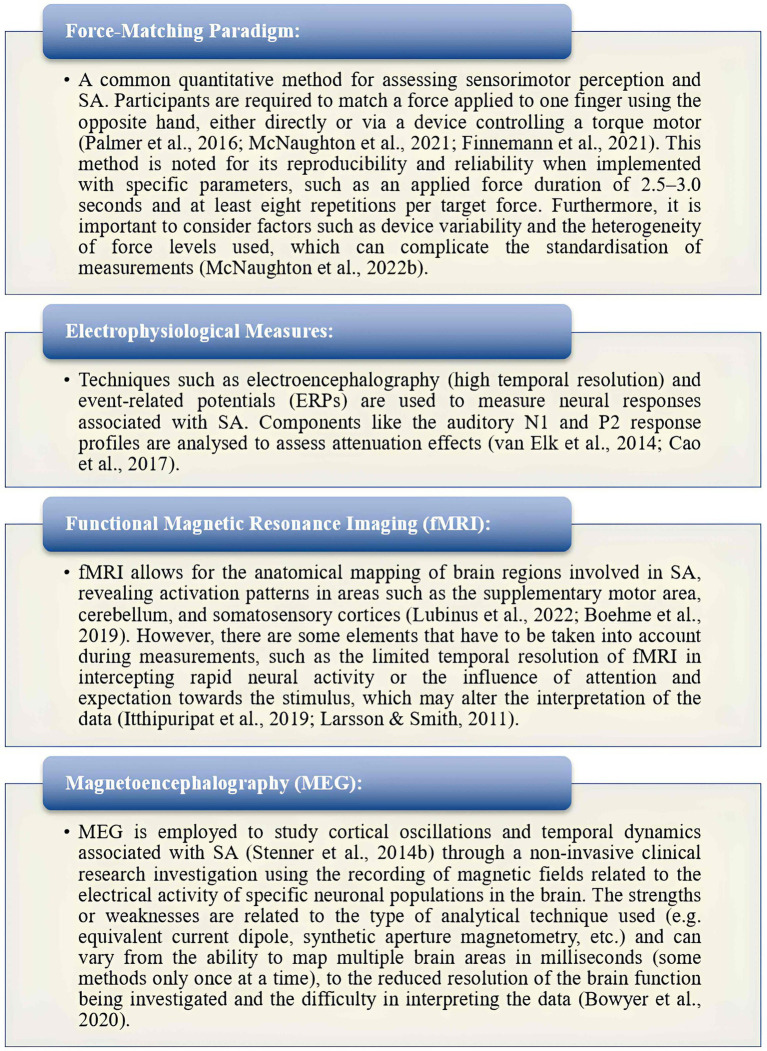
Measurement of sensory attenuation (Larsson and Smith, 2011; Itthipuripat et al., 2019; Bowyer et al., 2020).

SA has been measured using various methods across the included studies (Figure 5).

##### 3.3.3.1 Implications and applicability

**Figure 6:**
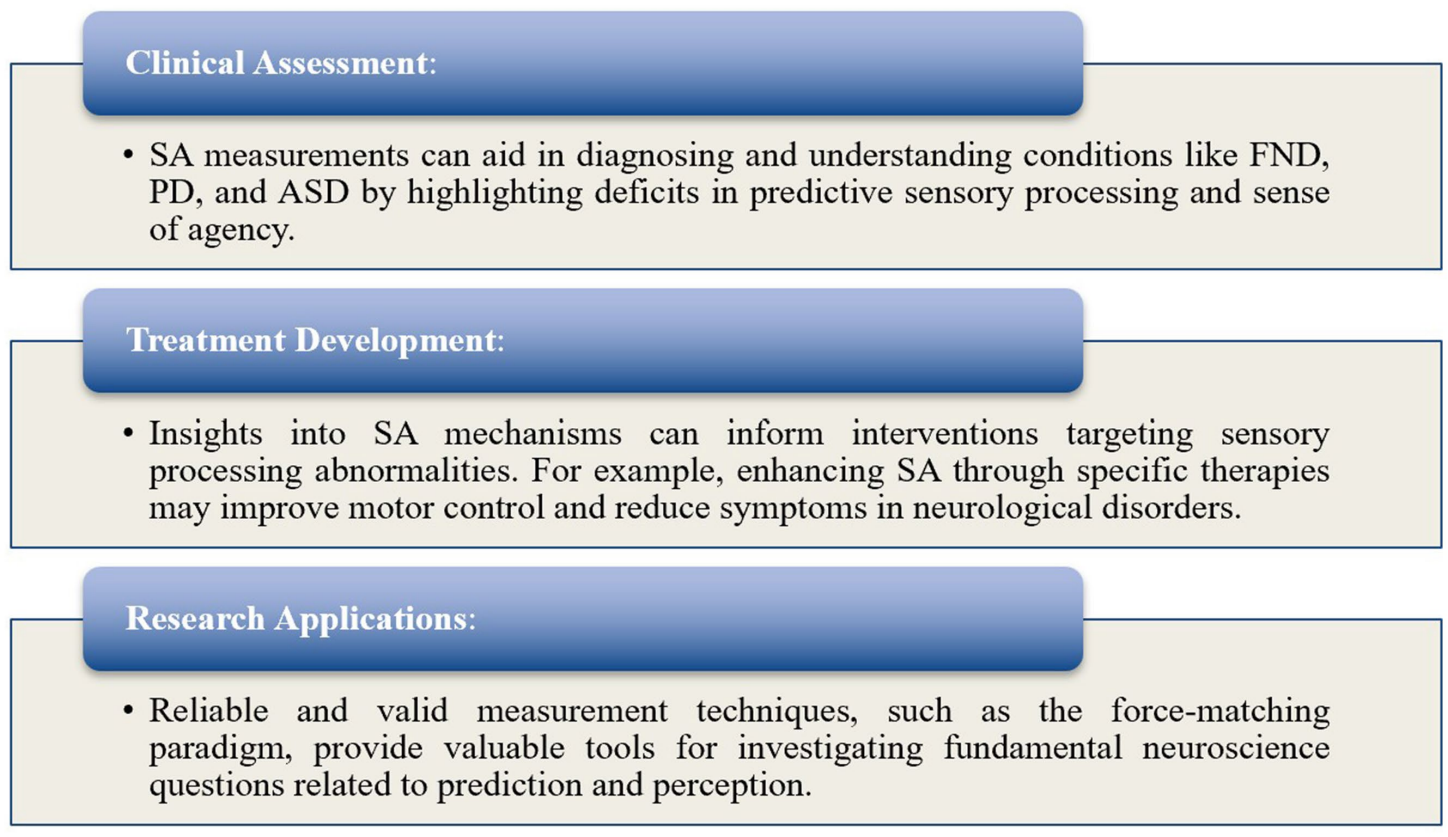
Implications and applicability of SA.

Understanding SA measurement techniques is crucial for elucidating the neural mechanisms underlying sensory processing in both healthy and symptomatic individuals (Figure 6).

### 3.4 Characteristics of included studies

Detailed characteristics of each included study are presented in Supplementary Table 1. This includes information on authors, year, origin, study design, aims, population demographics, intervention types, and key findings relevant to the scoping review questions.

## 4 Discussion

### 4.1 Interpretation and applications

The primary objective of this scoping review was to explore the role of SA in healthy individuals, FND, neurological disorders, and chronic pain. The secondary objective was to investigate how SA is measured in both healthy and symptomatic subjects and to assess the implications and applicability of these tests, particularly in individuals with PPS.

Our findings confirm that sensory attenuation is a fundamental neurophysiological mechanism by which the brain filters self-generated sensory information, prioritising externally generated, potentially salient stimuli. In healthy individuals, SA facilitates the differentiation between self-initiated actions and external events, playing a crucial role in the development of a sense of agency (SoA) and efficient sensorimotor integration (Csifcsák et al., 2018; Parthasharathy et al., 2022). This process allows for seamless interaction with the environment, wherein irrelevant self-generated stimuli are filtered out, enabling efficient and adaptive responses to non-self-generated stimuli.

In contrast, in individuals with FND and other neurological disorders, such as PD and HD, alterations in sensory attenuation have been observed. For example, patients with FND demonstrate reduced SA, potentially impairing their ability to recognise self-generated movements, which may contribute to the development and persistence of their symptoms (Pareés et al., 2014; Macerollo et al., 2015). Similarly, research has shown that dopaminergic treatment in PD can modulate SA, potentially enhancing sensory and motor prediction accuracy (Wolpe et al., 2018). These findings suggest that deficits in SA play a critical role in the functional and perceptual abnormalities observed in these conditions.

Chronic pain represents another domain where alterations in SA are particularly relevant. The evidence indicates that individuals with chronic pain exhibit disruptions in SA mechanisms, which is likely to lead to heightened pain perception and increased sensory sensitivity (McNaughton et al., 2022a; Lalouni et al., 2021). When SA is compromised, the nervous system’s ability to filter out irrelevant sensory inputs is diminished, resulting in an exaggerated awareness of nociceptive and even non-nociceptive signals leading to symptoms such as hyperalgesia and allodynia, respectively. This over-attention to sensory input perpetuates the experience of pain, reinforcing a maladaptive cycle of hypervigilance and symptom fixation (Esteves et al., 2022). Thus, chronic pain and other PPS can be understood as a consequence of the brain’s impaired capacity to modulate sensory information, making SA a crucial target for therapeutic intervention.

In terms of measurement, various techniques have been employed to assess SA, offering insights into how this process functions across different conditions. The force-matching paradigm, a widely used behavioural task, has consistently demonstrated that individuals tend to overestimate self-generated forces compared to externally applied ones (McNaughton et al., 2021). Additionally, neurophysiological techniques such as EEG, ERPs, fMRI, and MEG have been utilised to measure SA by examining brain responses to self-generated versus externally generated stimuli across multiple sensory modalities, including auditory, visual, and tactile domains (van Elk et al., 2014; Lubinus et al., 2022; Stenner et al., 2014b). These methods provide valuable insights into the supraspinal neurophysiological alterations associated with SA, especially in symptomatic subjects, such as those with PPS.

In patients with FND, altered SA may reflect disrupted predictive processing, contributing to the persistence of their symptoms (Pareés et al., 2014). Similarly, assessing SA in chronic pain patients could help identify maladaptive sensory processing patterns that perpetuate pain experiences (Lalouni et al., 2021). This has significant implications for clinical practice, particularly in the management of chronic pain and PPS, where interventions that aim to normalise sensory processing can be highly beneficial.

### 4.2 Implications for practice

A comprehensive understanding of sensory attenuation and its underlying neurobiological mechanisms is essential for clinicians involved in musculoskeletal care, including osteopaths, physiotherapists, and chiropractors. Sensory attenuation refers to the brain’s ability to selectively filter and modulate sensory input, prioritising stimuli that are most relevant for generating further cognitive processing while ignoring less pertinent signals. In healthy individuals, this process allows the body to “disappear” from conscious awareness, maintaining a state of effortless interaction with the environment (Leder, 1990). However, in individuals with PPS, such as chronic pain, this sensory attenuation process becomes compromised, leading to an overwhelming influx of sensory information that the brain struggles to manage effectively (Esteves et al., 2022). This breakdown in sensory gating lies at the heart of many chronic musculoskeletal conditions and serves as a critical target for therapeutic intervention.

The failure of sensory attenuation in PPS manifests as an inability to filter out irrelevant sensory inputs, resulting in an exaggerated awareness of bodily sensations that would normally remain subliminal. For example, signals related to interoception, such as heartbeats, muscle tension and proprioceptive joint movements, become amplified, contributing to the perception of pain or discomfort even in the absence of an ongoing physical threat. This over-attention to sensory input not only intensifies the experience of pain but also disrupts the individual’s ability to engage with their environment, reinforcing a maladaptive cycle of vigilance and symptom fixation (Esteves et al., 2022). Thus, PPS can be understood as a breakdown in the nervous system’s capacity to effectively attenuate and regulate sensory information.

From a neurobiological perspective, sensory attenuation is mediated by processes such as synaptic gain control, neuroplasticity and altered neuromodulatory mechanisms that adjust the precision or weighting of sensory signals (Friston, 2009). When these mechanisms are impaired, the brain’s ability to filter out irrelevant sensory information is diminished, leading to a state of constant vigilance and discomfort. This impairment can explain why individuals with chronic pain are unable to attend away from their symptoms or “turn down” the volume of sensory input, as their nervous (and associated systems see Kiverstein et al., 2022) fail to gate sensory evidence appropriately. This breakdown in sensory attenuation often results in an overwhelming and distressing influx of sensations, leading to heightened sensitivity, hypervigilance, and the perpetuation of PPS (Friston and Frith, 2015; Edwards et al., 2012).

In this context, the dyadic therapeutic relationship plays a pivotal role in helping patients recalibrate their sensory processing. By engaging in a shared therapeutic space, clinicians can facilitate the process of re-learning how to attenuate sensory inputs. The therapist-patient interaction serves as an opportunity to synchronise attentional focus and sensory processing, enabling the patient to regain control over their sensory experiences. This synchronisation is not merely a metaphorical connection but a tangible interaction involving physical touch, posture, verbal communication, and affective engagement, which are all crucial components in guiding the patient’s attention away from maladaptive sensory patterns (Esteves et al., 2022).

The dyadic therapeutic process offers a unique opportunity to restore effective sensory attenuation by creating a shared narrative and sensorium. This means that, within the therapeutic relationship, both the patient and the therapist contribute to a synchronised experience where sensory evidence can be reinterpreted and re-weighted. For example, through affective touch and other forms of hands-on therapy aided by careful language and demonstrated empathy, the therapist can help the patient experience sensations in a different, less threatening context. This process allows for the exploration of alternative narratives around pain and discomfort, thereby facilitating the re-establishment of sensory attenuation. By experiencing their bodily sensations in the presence of a trusted therapist, patients can begin to understand that not all sensory input requires heightened attention or response, gradually learning to filter out unnecessary signals once again (Vasil et al., 2020).

Moreover, this dyadic engagement aligns with predictive processing and active inference models, where the deployment of attention is understood as a form of covert action that adjusts the weighting of sensory evidence (Friston and Frith, 2015). Within this framework, chronic pain can be seen as a hypothesis that is repeatedly verified by selective attention to interoceptive and nociceptive signals. The role of the therapist, therefore, is to help the patient challenge this hypothesis by directing attention towards alternative sources of sensory evidence. This process helps to shift the patient’s perception of their symptoms, enabling them to develop a more adaptive sensory attenuation mechanism.

Engaging patients in active therapies that involve movement and sensory feedback, such as sensorimotor retraining, graded motor imagery, and attentional modulation techniques, can further support the recalibration of altered SA processes. For example, mirror therapy and virtual reality can provide congruent visual and proprioceptive feedback, allowing patients to recalibrate sensory predictions and improve the balance between expected and actual sensory input (Kilteni and Ehrsson, 2017). These methods can help modify maladaptive generative models by providing patients with opportunities to experience congruent and non-threatening sensory stimuli, which over time can contribute to reducing prediction errors and improving functional outcomes.

The breakdown of sensory attenuation in PPS also underscores the importance of incorporating physical touch and interaction in musculoskeletal care. When patients experience pain, they frequently become hyper-focused on specific body parts, leading to a narrowing of their attentional field. Engaging in affective touch, such as gentle palpation or rhythmic movement, provides a form of sensory input that is less threatening and more predictable. This allows the patient to experience their body in a way that is not dominated by pain, facilitating the recalibration of sensory attenuation. For instance, by synchronising touch with the patient’s breathing or heart rate, the therapist can help the patient establish a more balanced and regulated sensory experience, promoting the attenuation of irrelevant or distressing sensory signals (Esteves et al., 2022).

Furthermore, educating patients about the role of sensory attenuation and its impact on their symptoms can empower them to take an active role in their rehabilitation. Understanding that altered sensory processing contributes to their experiences can help to alleviate anxiety and improve adherence to therapeutic interventions, ultimately supporting the restoration of effective sensory gating. This educational component reinforces the idea that sensory experiences can be modulated, offering patients a sense of agency over their symptoms.

Ultimately, this dyadic approach to sensory attenuation and PPS enables a more holistic and effective form of treatment. By leveraging the therapist-patient relationship to foster a shared sensory experience, clinicians can guide patients towards regaining control over their sensory processing, thereby addressing the core dysfunctions that sustain chronic pain and other related conditions. This integrative approach aligns with modulatory theories of pain but extends beyond it, offering a comprehensive framework that addresses the root of sensory dysregulation. As Esteves et al. (2022) emphasise, the therapeutic process should focus not only on altering the patient’s sensory input but also on transforming their capacity to modulate and attenuate these inputs, ultimately restoring the body’s ability to “disappear” into the background of conscious experience.

By emphasising sensory attenuation within the context of a dyadic therapeutic relationship, clinicians can address the fundamental challenges of PPS. This approach provides a pathway for patients to re-learn how to gate sensory information effectively, ultimately reducing their experience of pain and discomfort and restoring a more adaptive engagement with their bodies and environment. It is this shared therapeutic journey, grounded in sensory synchronisation and attenuation, that offers the most promising avenue for fostering long-term recovery and well-being in individuals with chronic musculoskeletal conditions. This integration of sensory attenuation strategies into manual therapy and musculoskeletal care practices stands as a critical and innovative approach for improving patient outcomes.

Integrating SA-based interventions into manual therapy offers a novel pathway to modulate predictive coding mechanisms and improve clinical outcomes in patients with PPS. Manual therapy inherently provides controlled tactile and proprioceptive input, making it an ideal platform for modifying sensory precision estimates and reducing prediction error within sensorimotor systems (Esteves et al., 2022). Techniques such as affective touch, passive joint mobilisation, or rhythmic soft tissue work may enhance sensory attenuation by providing consistent, low-threat input that aligns with patient expectations and reinforces adaptive sensorimotor models (Kim et al., 2022; McParlin et al., 2023). Additionally, coupling manual therapy with explicit attentional guidance or verbal reappraisal can further engage top-down mechanisms that regulate sensory filtering (Cerritelli and Esteves, 2022). Emerging approaches suggest that layering manual therapy with technologies like virtual reality (VR) or real-time feedback from wearable sensors may further amplify SA by reinforcing sensory congruency. This integrative framework aligns with active inference principles and could help “retrain” the predictive brain toward more adaptive interpretations of bodily signals in functional and chronic pain syndromes (Bohlen et al., 2021).

### 4.3 Strengths and limitations

This review offers a comprehensive examination of SA across different populations and sensory domains, providing a broad perspective on its role in health and disease. By including both healthy and symptomatic subjects, we have highlighted how SA functions across a continuum and how disruptions in this process can contribute to various conditions, particularly FND and chronic pain.

While our review acknowledges the considerable heterogeneity in study populations, experimental paradigms, and measurement techniques used to assess sensory attenuation (SA), we recognise that this variability complicates the synthesis of findings and limits generalisability. To address this, we now propose a structured synthesis framework that stratifies findings across three axes: (1) clinical population (e.g., FND, chronic pain, PD), (2) sensory modality assessed (e.g., tactile, auditory, visual), and (3) methodology used (e.g., force-matching, EEG/ERP, fMRI). This approach allows for a clearer mapping of consistencies and divergences within and between domains. For instance, force-matching studies consistently reveal reduced SA in chronic pain populations but are less frequently applied in psychiatric cohorts, while EEG paradigms often highlight attenuated N1 components in FND but with differing baselines across tasks (Wolpert et al., 2011). By structuring the literature along these axes, researchers can better identify gaps, methodological biases, and candidate targets for standardisation. This strategy aligns with recent calls to adopt transdiagnostic research frameworks and modality-specific mappings to overcome the limitations of small, siloed datasets and foster cumulative science in SA research. The complexity of SA and its underlying neural mechanisms suggests that interpretations should be approached with caution. Additionally, practical applications of SA assessments in clinical settings remain a challenge, as many techniques, such as fMRI and MEG, are not readily accessible in routine practice. Although the force-matching paradigm offers valuable insights into SA, its application in clinical settings is limited due to the need for specialised equipment and time constraints.

Future research should focus on developing accessible and reliable clinical tools to assess SA that can be readily integrated into therapeutic settings. This would allow clinicians to more effectively identify and address disruptions in SA among individuals with chronic pain and other PPS, enhancing the efficacy of treatment interventions.

## 5 Conclusion

This scoping review highlights the pivotal role of SA in filtering self-generated sensory inputs from external stimuli, particularly in chronic pain and PPS. Altered SA significantly contributes to symptoms by leading to heightened sensitivity and maladaptive fixation on symptoms.

Understanding SA offers valuable insights for manual therapy and MSK care. Recognising that disruptions in SA underlie many chronic pain experiences, clinicians can adopt person-centred approaches to recalibrate sensory processing through interventions like sensorimotor retraining, attentional modulation, and hands-on therapies leveraging affective touch. These strategies align with active inference models to update maladaptive generative models and re-establish effective SA.

The therapeutic relationship is crucial in addressing sensory dysfunctions. By fostering a shared therapeutic space, clinicians guide patients in modulating sensory inputs, helping them regain control over their sensory processing and reduce symptom burden.

Future research should focus on standardising clinically feasible SA measurement techniques and exploring how therapeutic interventions can modulate SA to refine treatment approaches. Investigating SA’s role across different PPS populations may lead to innovative, evidence-based strategies within the active inference framework, enhancing the efficacy of MSK care.

To enhance the translational value of sensory attenuation research, we propose several concrete avenues for future tool development and standardisation. First, the adaptation of force-matching paradigms into app-based platforms using haptic-feedback-enabled tablets or wearable sensors would facilitate bedside and remote assessments. These technologies can quantify sensorimotor prediction errors with high ecological validity and minimal training. Second, portable EEG systems, increasingly used in neurofeedback and brain-computer interface research, hold promise for implementing low-cost SA-related ERP markers (e.g., N1 suppression) in outpatient settings. Third, multimodal integration frameworks that combine behavioural (e.g., reaction time, force perception) and physiological (e.g., EEG, EMG) measures could improve both diagnostic precision and inter-subject comparability. Developing consensus protocols for these hybrid models will be crucial for reproducibility. Finally, clinical trials should evaluate these tools not only as diagnostic endpoints but as therapeutic probes—assessing whether SA-targeted interventions (e.g., active inference-informed sensorimotor retraining, VR immersion) induce measurable changes in SA and symptom severity. Standardisation of SA assessments, including normative data across populations, will be critical to integrating SA markers into stratified care and neurorehabilitation models.

In summary, integrating SA strategies into manual therapy and MSK care presents a promising approach for improving patient outcomes. By addressing sensory dysregulation and fostering collaborative therapeutic relationships, clinicians can provide more effective treatments, enabling individuals with chronic musculoskeletal conditions to achieve long-term recovery and well-being. This approach not only reaffirms the importance of SA in health and disease but also opens avenues for advancing patient-centred care, ensuring that therapeutic interventions are grounded in an understanding of the complex interplay between sensory processing, perception, and the experience of pain.
