# Supplementary material for: The role of sensory attenuation in symptomatic and healthy individuals: a scoping review
Source: Front Neurosci. 2025 Jun 23;19:1590127. doi: 10.3389/fnins.2025.1590127 (PMC12230077; doi:10.3389/fnins.2025.1590127)
Supplement: Supplementary file 1 [file Table_1.docx]

Supplementary Table 1: Details of the studies included

| **Authors (Year)** | **Country / Study Design / Aims** | **Population / Sample Size / Age in years (M & SD) / Sex** | **Intervention Type** | **Key Findings Related to the Scoping Review Question(s)** |
| --- | --- | --- | --- | --- |
| **Storch & Zimmermann (2022)** | Germany / Experimental / Influence of predictable temporal stimuli on SA | 30 participants / M: 26; SD (no) / 11 men, 19 women | Judged orientation of flashing Gabor patches relative to button pressing in varied temporal conditions | Temporal attention and SA are independent; SA can be modulated by temporal predictability |
| **Bolt & Loehr (2021)** | USA / Experimental / Examine SA in joint action and auditory potentials | 80 participants / M: 22y; SD: 4.1 / 29 men, 51 women | Participants produced tone sequences alone and in pairs, generating self- or partner-generated sounds | No SA observed in auditory N1 for self- or partner-generated sounds; SA observed in auditory P2 during synchronous action for self-generated sounds |
| **Knoetsch & Zimmermann (2021)** | Germany / Experimental / Determine spatial selectivity of SA | 25 participants / M: 25.83; SD: 2.04 / 11 men, 14 women | Tactile impulses delivered to index or ring finger, triggered by self or delayed button press | SA for self-generated touch is spatially selective; determined by correlation between movement and sensory effects |
| **McNaughton et al. (2022a)** | Australia / Cross-sectional / Investigate SA's role in chronic pain perception | 131 participants (66 chronic pain, 65 controls) / M-chronic pain: 24.95; SD: 8.86; M-controls: 23.88; SD: 8.74 / 40 men, 91 women | Strength-matching task and questionnaires assessing pain and psychological factors | No significant differences in SA magnitude between groups; chronic pain patients showed greater tactile variability in SA |
| **Palmer et al. (2016)** | UK / Experimental / Link between physiological SA and perceptual SA using force-matching task | 18 participants / M: 28.24; SD: 8.53 / 9 men, 9 women | Median nerve stimulation during force reproduction tasks with EEG recordings | Physiological SA (neural responses) and perceptual SA (behavioural responses) exhibit different functional activities |
| **Macerollo et al. (2015)** | UK & Italy / Experimental / Examine SA in functional movement disorders (FMD) | 34 participants (17 FMD patients, 17 controls) / M-FMD patients: 45.5y & SD: 7.6; M-control: 48 & SD 7.4 / 12 men, 22 women | Median nerve stimulation at rest and during self-paced thumb movement | FMD patients show reduced SA compared to healthy controls |
| **Harrison et al. (2021)** | Australia / Experimental / Effects of temporal predictability and control on SA | Exp 1: 42 participants / M: 20.69; SD: 3.71 / 17 men, 25 women Exp 2: 38 participants / M: 21.38; SD: 4.80 / 13 men, 25 women | Visual tasks with auditory stimuli generated actively or passively under different timing conditions | Self-generated sounds elicit lower cortical activity; SA is influenced by predictability and temporal control |
| **Brown et al. (2013)** | UK / Review / Relationship between agency, active inference, and SA | N/A | Theoretical analysis of SA in relation to agency and motor control | Agency attribution involves attenuating attention to self-produced sensations; implications for functional movement disorders |
| **Kearney & Brittain (2021)** | UK / Review / Role of SA in Parkinson's disease (PD) | N/A | Review of SA mechanisms in PD and implications for rehabilitation | PD patients show less differentiated responses to stimuli; suggests difficulty in integrating sensory inputs and producing movements |
| **Wolpe et al. (2018)** | UK / Experimental / Abnormalities in voluntary movement initiation in PD | 193 participants (18 PD patients, 175 controls) / M-PD patients: 67 & SD: 10; M-controls: 65 & SD: 10 / 101 men, 92 women | Force-matching task assessing SA before and after dopaminergic medication | No significant differences in SA between medicated PD patients and controls |
| **Hua et al. (2020)**  **11** | UK / Experimental / Neural networks underlying SA mechanism of the M100 auditory response | 35 participants / M: 22.3; SD: (?) / 9 men, 26 women | Auditory stimuli with active button press or passive listening, EEG recordings | Multiple cortical areas involved in SA; supports predictive processing in sensory and motor contexts |
| **Han et al. (2021)** | Australia / Experimental / SA under complete control of auditory stimulus onset | Exp 1: 42 participants / M: 22; SD: 4.3 / 21 men, 21 women Exp 2: 47 participants / M: 20.3; SD: 5.6 / 18 men, 29 women | Decision-making task with optional button press affecting sound presentation timing | Motor actions and sense of control affect evoked responses differently; distinct elements of auditory potentials are involved |
| **Lee & Schmit (2018)** | USA / Experimental / Sensory deficits on movement-related cortical fluctuations | 10 participants / Age range: 20–35 / M: no; SD: no / 5 men, 5 women | Electrical stimulation of nerves and ankle exercises under various conditions | Attenuation of sensory feedback decreases beta-band synchrony; reflects mechanisms of sensorimotor integration |
| **Pinto et al. (2021)** | Belgium / Experimental / Does SA extend to pain perception? | Exp 1: 68 participants / M: (?); SD: (?) / 26 men, 42 women Exp 2: 79 participants / M: (?); SD: (?) / 18 men, 61 women | Painful stimuli delivered during motor execution, preparation, or rest; intensity evaluations | Pain processing influenced by motor execution, not preparation; suggests pain perception is modulated by actions |
| **Porciuncula et al. (2020)** | USA / Observational / Effect of SA on postural control in Huntington's disease (HD) | 39 participants (17 pHD, 11 mHD, 11 controls) / M-control: 43.8 & SD: 11.4; M-pHD: 41.1 & SD:9.3; M-mHD: 50 & SD: 11.8 / Sex not specified | Measurements of sway during postural tasks with varying sensory demands | Manifest HD patients show postural deficits increasing with sensory demand; pre-manifest patients responsive to sensory enhancement |
| **Hughes (2015)** | UK / Experimental / Neurophysiological and behavioral effects of emotional expressions on SA | 23 participants / Mean age: 22 and 10 months; SD: (no) / 9 men, 14 women | Finger-press tasks with EEG recordings, associating stimuli with "fear" or "not fear" words | Increased SA for stimuli with high social relevance; social and affective outcomes influence SA and sense of agency |
| **Burin et al. (2017)** | Italy / Within-subjects / Role of body reafferences in SA beyond feedforward motor signals | 12 participants / Mean age: 21.96; SD: (no) / 6 men, 7 women | Nerve stimulation with varying types; assessments using Likert scales | Reafferent body signals modulate SA; indicates SA is influenced by feedback from the body |
| **Ciaunica et al. (2022)** | Portugal / Review / Pathophysiology of depersonalization disorder through predictive processing | N/A | Theoretical analysis of SA in depersonalization disorder | Suboptimal SA may hinder self-awareness sharpening; close physical interactions may alleviate symptoms |
| **Finnemann et al. (2021)** | UK / Experimental / Temporal link between SA and agency in ASD | Exp 1: 50 participants (24 ASC, 26 controls) / M-ASC: 30.1 & SD: 9.2; M-Controls: 30.6 & SD: 6.0) / 20 men, 30 women; Exp 2: 48 participants (23 ASC, 25 controls) / M-ASC: 29.0 & SD: 6.1; M-Controls: 25 & SD: 5.7 / 21 men, 27 women | Force-matching task and temporal perception tasks comparing ASD and controls | No general deficit in predictive processing in ASD; intact SA mechanisms observed |
| **Stenner et al. (2014a)** | UK / Experimental / Attribution of SA to motor processes | 16 participants / M: 22.8; SD: 3.2 / 8 men, 8 women | Forced-choice tasks involving button presses and tone discrimination | SA of action consequences is guided by top-down motor processing |
| **Stenner et al. (2014b)** | UK / Experimental / Parallel processing of motor output and sensory prediction | 17 participants / M: 25.4; SD: 5.4 / 8 men, 9 women | Similar to previous study; focus on sensory cortex modulation during action preparation | Anticipatory modulation in sensory cortex occurs independently of stimulus expectation or attention |
| **Cao et al. (2017)** | UK / Experimental / Role of brain oscillations in auditory SA | 14 participants / M: 22.6; SD: 1.8 / 6 men, 8 women | Auditory stimuli under passive and active conditions; EEG recordings | Low-frequency auditory oscillations involved in mitigating SA effects; supports hierarchical neural regulation |
| **Hua et al. (2023)** | UK / Experimental / Auditory SA deficits in early psychosis | 180 participants (109 CHR-P, 48 HC, 23 FEP) / M-CHR-P: 22.0 & SD: 4.5; M-HC: 22.8 & SD: 3.6; M-FEP: 23.2 & SD: 3.5 / 57 men, 123 women | Auditory tasks with active and passive conditions; EEG recordings | Early psychosis characterized by SA changes in auditory and thalamic regions |
| **Lalouni et al. (2021)** | Sweden / Experimental / Does SA occur in painful sensations? | 40 participants / M: 25.1; SD: 4.5 / 18 men, 21 women, 1 non-binary | Self-applied and externally applied pressure using an algometer; pain threshold assessments | SA occurs in both self-generated and imagined conditions; increased pain threshold observed |
| **McNaughton et al. (2022b)** | Australia / Experimental / Assessment of force-matching task protocols | 138 participants / M: 24.51; SD: 8.71 / 43 men, 95 women | Direct and slider trials of the force-matching task with varying force levels | Force-matching task effectively measures SA; recommended protocol parameters provided |
| **Lubinus et al. (2022)** | Germany / Within-subjects / SA phenomena in visual domain; role of efference copies vs. time prediction | 25 participants / M: 23.8; SD: 2.2 / 10 men, 15 women | Visual stimuli presented with predictable or unpredictable timing relative to self-generated actions | SA in visual domain based on efference copies rather than time predictions |
| **Richter & de Lange (2019)** | Netherlands / Experimental / Modulation of SA by attention following visual statistical learning | 34 participants / M: 24.9; SD: 4.8 / 9 men, 25 women | Visual tasks with predictive stimuli sequences; attention manipulation; MRI sessions | SA occurs for predicted inputs only when processed with attention; attention directs integration of prior knowledge and sensory input |
| **Adams et al. (2013)** | Switzerland / Review / Psychotic symptoms as inference or belief errors | N/A | Theoretical exploration of predictive coding in psychosis | Schizophrenia may involve decreased anticipatory accuracy or lack of sensory attenuation |
| **Csifcsák et al. (2018)** | Norway & Hungary / Experimental / Action-related prediction mechanisms on visual evoked potentials | Exp 1: 17 participants / Mean age: 24.0; SD: 3.8 / 8 men, 9 women; Exp 2: 19 participants / Mean age: 25.7; SD: 6.0 / 9 men, 10 women | Visual stimuli under passive, motor induction, and motor-only conditions; EEG recordings | Visual attenuation observed; findings may help understand psychiatric conditions like schizophrenia |
| **Gentsch et al. (2015)** | UK / Experimental / Sensory experience during active caressing of self vs. others | 133 pairs of participants / M: 23.4; SD: 4.2 / women | Participants caressed their own or another's forearm; sensory evaluations using VAS | Active stroking of others increases perceived sensory experience compared to self-touch |
| **Pareés et al. (2014)** | UK / Experimental / SA in FND using force-matching task | 28 participants (14 FND patients, 14 controls) / M-FND: (Only median) & SD: (No); M-Controls: (Only median) & SD: (No) / 5 men, 23 women | Force-matching task comparing self-generated and externally generated forces | FND patients overestimated force less than controls; suggests loss of SA related to altered sense of agency |
| **Abbasi & Gross (2019)** | Germany / Experimental / Motor-auditory cortical connectivity during SA | 18 participants / Mean age: 26.6; SD: (3.9?) / 9 men, 9 women | Active and passive auditory tasks with EEG recordings | Functional connectivity between motor and auditory areas underlies SA; reflects internal prediction mechanisms |
| **van Laarhoven et al. (2019)** | Netherlands / Experimental / Auditory SA in ASD | 60 participants (30 ASD, 30 TD) / M-ASD: 18.55 & SD: 2.13; M-TD: 18.83 & SD: 1.32 / 46 men, 14 women | Auditory tasks with self-generated and externally generated sounds; EEG recordings | Individuals with ASD show impaired SA for self-generated sounds; supports altered predictive processing |
| **McNaughton et al. (2021)** | Australia / Experimental / Mechanical assessment of haptic devices in force-matching tasks | 25 participants / Mean age: 30.8; SD: (?) / 12 men, 13 women | Participants used haptic devices to perform force-matching tasks | Device effectively detects SA levels; provides a behavioural measure of SA |
| **Roussel et al. (2014)** | UK / Experimental / Relationship between neurophysiological and psychophysical measures in SA | 15 participants / Mean age: 24; SD: 3.69? / 7 men, 8 women | Self-generated movements with letter discrimination tasks; EEG recordings | Reduced sensory processing of voluntary action consequences observed both behaviourally and neurally |
| **Vasser et al. (2019)** | Estonia / Experimental / Mechanisms underlying neurophysiological processes of SA | Exp 1: 8 participants / M: 25; SD: no / 6 men, 2 women; Exp 2: 46 participants / M: 23; SD: no / 19 men, 27 women | Visual tasks with Gabor patches in virtual reality; assessments of sensitivity and confidence | SA affects first-order sensitivity but not second-order metacognitive judgments |
| **Cao & Gross (2015)** | UK / Experimental / Cultural differences in processing self- vs. externally-generated sounds | Chinese group: 30 participants / M: 22.4; SD: 1.7 / 15 men, 15 women;  British group: 30 participants / M: 21.9; SD: 1.7 / 15 men, 15 women | Auditory tasks with self-generated and externally generated sounds; volume assessments | Cultural differences observed; collectivist cultures (Chinese) show SA for others' actions, individualist cultures (British) show SA linked to self-construal |
| **Dogge et al. (2019)** | Netherlands / Experimental / Role of motor predictions in SA and learning effects | 24 participants / M: 20.63; SD: 2.45 / 9 men, 15 women | Auditory discrimination tasks with learned associations between actions and sounds | SA is more pronounced when participants rely on motor predictions; learning influences SA effects |
| **van Elk et al. (2014)** | Switzerland & Netherlands / Experimental / Auditory SA for sounds generated by appendicular movements | 12 participants / M: 22.0; SD: no / 8 men, 4 women | Participants pressed buttons with hands and feet, triggering sounds; EEG recordings | SA reflected in N1 attenuation for self-generated sounds from limbs; supports embodiment in action perception |
| **Loehr (2013)** | Canada / Experimental / SA in jointly produced tones | 48 participants / Mean age: 22.71; SD: 3.88 / 10 men, 38 women | Participants produced tones alone or jointly with others; auditory potentials measured | SA for N1 is lower for jointly generated sounds; individuals discriminate their own contribution in joint actions |
| **Fritz et al. (2022)** | Germany / Experimental / Does tactile attention migrate toward the contact area during movement? | Exp 1: 29 participants / M: 26.52; SD: 9.87 / 11 men, 17 female, 1 non-binary Exp 2: 25 participants / M: 24.46; SD: 9.14 / 6 men, 19 women Exp 3: 20 participants / M: 32.55; SD: 14.34 / 9 men, 11 women | Participants performed hand movements in virtual reality with tactile and auditory stimuli | SA temporarily increases tactile sensitivity during hand movements toward a target |
| **Orepic et al. (2021)** | Switzerland / Randomized block design / SA in auditory domain producing psychosis-like phenomena | Exp 1: 30 participants / M: 21.8; SD: 2.4 / 9 men, 21 women Exp 2: 30 participants / M: 23.7; SD: 2.4 / 14 men, 16 women | Participants engaged in conversations with altered auditory feedback; assessments of psychosis-like experiences | Sensory and motor stimulation can induce psychosis-like states; individuals with schizophrenia show reduced self-attention |
| **Pyasik et al. (2021)** | Italy / Within-subject / Behavioral and neurophysiological processes of SA in somatosensory stimuli | 14 participants / Details not specified / 5 men, 9 women | Rubber hand illusion paradigm combined with SA tasks | Body ownership significantly affects perceived sensations; SA is modulated by embodiment |
| **Parthasharathy et al. (2022)** | Belgium / Experimental / SA near the upper extremity and aging effects | Exp 1: 70 participants (35 young adults, 35 older adults) / Details not specified; Exp 2: 61 participants (31 young adults, older adults) / Details not specified | Force-matching tasks using a robotic arm; assessments of SA across ages | SA is greater in individuals over 55; may be due to proprioceptive deficits with aging |
| **Kilteni & Ehrsson (2017)** | Sweden / Experimental / Relationship between body ownership and SA using predictive models | Exp 1: 24 participants each / Details not specified / 9 men, 15 women  Exp 2: 24 participants each / Details not specified / 11 men, 13 women | Force-matching tasks with and without a rubber hand illusion | Sense of body ownership determines SA; body representation updates influence sensory predictions |
| **Fritsch et al. (2021)** | Germany / Proof-of-concept / SA with prosthetic devices in amputees | 13 amputees using prosthetic devices / M: 60.31; SD: 8.75 / 12 men, 1 woman | Participants made judgments on sensory experiences when touching with intact hand, prosthetic hand, or being touched by others | Prosthetic devices can be embodied; SA occurs with prosthetic use similar to intact limbs |
| **Fritz & Zimmermann (2023)** | Germany / Experimental / Temporal dispersion analysis of temporal recalibration in SA | Exp 1: 36 participants / M: 25.63; SD: no / 15 men, 21 women Exp 2: 38 participants / M: 24.79; SD: no / 16 men, 21 women, **1 diverse** Exp 3: 36 participants / M: 24.66; SD: no / 9 men, 27 women | Self-touch tasks with varying delays between action and sensation | Temporal selectivity of SA results from single delay introduction; multiple delays lead to broader temporal spread of SA |
| **Kiepe et al. (2021)** | Germany / Mini Review / SA and its sensory meaning and function in behavior | N/A | Review of current knowledge on SA mechanisms | SA results from attention orientation based on anticipation; not solely derived from motor activity |
| **Windt et al. (2014)** | Germany / Hypothesis and theory / Possibility of tickling in different conscious states | Part 1: 61 participants / Details not specified Part 2: 9 participants / M: 20.7; SD: no / 5 men, 4 women | Participants engaged in real and imagined tickling during wakefulness and lucid dreaming; questionnaires used | Participants could not tickle themselves; suggests limitations in self-generated sensations across conscious states |
| **Boehme et al. (2019)** | Sweden / Experimental / Neural correlates of self-generated vs. external touch | Study 1: 27 participants / M: 23.4; SD: ? / 13 men, 14 women;  Study 2: 17 participants / M: 27.3; SD: ? / 8 men, 9 women;  Study 3: 10 participants / M: 27.7; SD: ? / 4 men, 6 women | Participants experienced self-touch, other-touch, and object-touch; fMRI, behavioral tests, and SEPs recorded | Self-touch results in different activation patterns; externally generated touch activates social and cognitive brain areas |
| **Boehme & Olausson (2022)** | Sweden / Review / SA and importance of self-generated touch | N/A | Review of SA mechanisms in touch | SA in touch may be due to anticipatory processes; challenges the view of global inhibition in self-touch |
| **Weller et al. (2017)** | Germany / Experimental / Relationship between SA and personal agency | 16 participants / M: 27.56; SD: no / 8 men, 8 women | Tasks involving button presses with immediate or delayed auditory feedback; sense of agency assessments | Partial dissociation between SA and sense of agency; postdictive processes contribute to agency perception |
| **Mifsud et al. (2018)** | Australia / Experimental / SA in visual domain using saccades paradigm | Exp 1: 11 participants / M: 19; SD: 1 / 5 men, 6 women Exp 2: 33 participants / M: 22; SD: 6 / 18 men, 15 women Exp 3: 25 participants / M: 19; SD: 6 / 12 men, 13 women | Visual stimuli presented with self-initiated saccades or external triggers; EEG recordings | SA observed for visual stimuli; electrophysiological attenuation linked to motor-sensory correspondence |
| **Dong & Bao (2021)** | China / Experimental / SA during preparation of head rotation movements | Exp 1: 8 participants / M: 22.13; SD: no / 2 men, 6 women;  Exp 2: 8 participants / M: 22.13; SD: no / 3 men, 5 women | Visual tasks requiring head movements; auditory stimuli presented at specific timings | Sensory component suppression occurs during movement preparation; attentional shifts play a significant role |
| **Nuruki et al. (2019)** | Japan / Experimental / Physiological processes in perceptual discrimination and SA | 14 participants / M: 22.5; SD: ? / 12 men, 2 women | Force-matching tasks with static and periodic force changes; assessments of SA | No significant differences in SA between static and periodic force changes; sensory feedback prediction functions adequately |
| **Jo et al. (2019)** | Germany / Experimental / Brain organization underlying prediction during SA | 39 participants / M: 40.5; SD: 7.4 / 14 men, 25 women | Intentional binding paradigm with button presses and auditory feedback; EEG recordings | Supplementary motor area involved in auditory prediction; contributes to controlled sensory predictions |
| **Kiepe et al. (2023)** | Germany / Experimental / Effects of virtual occlusion on SA | 29 participants / M: 23; SD: 4 / 7 men, 22 women | Hand movement tasks in virtual reality with occluded or visible hands; visual discrimination assessments | Virtual occlusion affects perceptual sensitivity; internal predictive signals modulate visual SA |
| **Klaffehn et al. (2019)** | Germany / Experimental / SA for self-initiated vs. external tones with temporal probability cues | 23 participants / M: 30.65; SD: 9.25 / 8 men, 15 women | Tasks involving button presses and tone presentations under different conditions | Voluntary actions involve unique predictive elements influencing perception of subsequent events |
| **Schwarz et al. (2018)** | Germany / Experimental / SA in visual field | Exp 1: 46 participants / M: 25.5; SD: ? / 8 men, 38 women Exp 2: 55 participants / M: 20.3; SD: ? / 8 men, 47 women Exp 3: 62 participants / M: 26.5; SD: ? / 11 men, 51 women | Visual tasks with Gabor patches following self-initiated actions; assessments of SA | No SA observed in visual domain across experiments; suggests SA is not universal or autonomous |
| **Mifsud & Whitford (2017)** | Australia / Experimental / N1 attenuation in self-generated vs. external auditory stimuli | 28 participants / M: 22; SD: 7 / 10 men, 18 women | Auditory tasks with self-initiated and externally initiated sounds; EEG recordings | N1 attenuation depends on correlation between initiating action and resulting sensation |
| **Hughes et al. (2013a)** | France / Experimental / SA related to action prediction | 16 participants / Mean age: 25 and 8 months; SD: no / 6 men, 10 women | Participants pressed keys linked to specific tones; predictability of action effects manipulated | SA arises from action-effect prediction; action shapes perception of external world |
| **Hughes et al. (2013b)** | France / Review / Analysis of SA and intentional binding mechanisms | N/A | Systematic review of SA and intentional binding studies | SA effects are partly caused by differences in time prediction; highlights importance of predictive processes |

Legend – Supplementary Table 1:

| Terminology | Acronyms |
| --- | --- |
| Sensory Attenuation | SA |
| Functional Neurological Disorders | FND |
| Functional Movement Disorders | FMD |
| Huntington's disease | HD |
| Premanifest Huntington's disease | pHD |
| Manifest Huntington's disease | mHD |
| Parkinson's disease | PD |
| Autism spectrum disorders | ASD |
| Autism spectrum conditions | ASCs |
| Typically Developing | TP |
| Clinical high-risk for psychosis | CHR-P |
| First-episode psychosis | FEP |
| Healthy controls | HC |
| Functional magnetic resonance imaging | fMRI |
| Electroencephalogram | EEG |
| Mean age | M |
| Standard Deviation | SD |
| Experiment | Exp |
