# Supplementary material for: The role of sensory attenuation in symptomatic and healthy individuals: a scoping review
Source: Front Neurosci. 2025 Jun 23;19:1590127. doi: 10.3389/fnins.2025.1590127 (PMC12230077; doi:10.3389/fnins.2025.1590127)
Supplement: Supplementary file 2 [file Data_Sheet_1.pdf]

# SUPPLEMENTARY MATERIAL

## VISUALISATION OF SCIENTIFIC LANDSCAPES

The VOSviewer 1.6.20 software was used to generate a network based on the textual data (title and abstract). Binary count, minimum number of occurrences (number of documents in which a keyword is present at least once) as term “2”, of 1468 terms 263 met the threshold. Of these, a relevance score was calculated and the system selected the most relevant terms (the default choice was to select 60% of the most relevant terms), resulting in 158 items, from which 77 other items were not significantly relevant to the creation of the network. The software classified the items into eight different clusters:

| Cluster & Number of items | Items |
|---|---|
| **Red (15)** | Inference, internal forward model, prediction error, auditory sensory attenuation, predictive coding framework, auditory cortex, brain, brain oscillation, failure, idea, precision, prediction process, predictive coding, psychosis, smaller brain response. |
| **Green (11)** | Perceptual sensory attenuation, tactile stimuli, touch, self-touch, social-touch, relationship, autism, sensorimotor cortex, social interaction, somatosensory evoked potential, thalamus. |
| **Blue (11)** | Discrimination, motor preparation, sensory suppression, execution, sensitivity, tactile sensation, target, visual domain, visual perception, visual sensitivity, suppression. |
| **Yellow (10)** | Temporal attention, temporal control, temporal prediction, action prediction, intentional binding, issue, schizophrenia, sensory processing, stimulus expectation, voluntary action effect. |
| **Purple (9)** | Body ownership, external world, movement disorder, pain, patient, pattern, reduced brain response, sensory response, voluntary movement. |
| **Green blue (9)** | Predictability, stimulus intensity, stimulus predictability, action consequence, external source, neural processing, perceptual effect, sensory action consequence, subliminal motor priming. |
| **Orange (9)** | Sensory attenuation paradigm, sensory information, ability, age, force, framework, Parkinson disease, proprioception, target force. |
| **Brown (7)** | Sensory event, human brain, self-generated tone, own action, joint action, goal, event related potential. |

Based on the clustering, two images were generated through the following methods:

- "Network Visualization" (see Figure 4): the label and circle size of an element are determined by the weight of the element. Specifically, the weight of the article is directly proportional to the size of the label and the circle of the item. The proximity of one element to another and the thickness of the lines connecting them are indicators of a strong correlation (van Eck & Waltman, 2023).

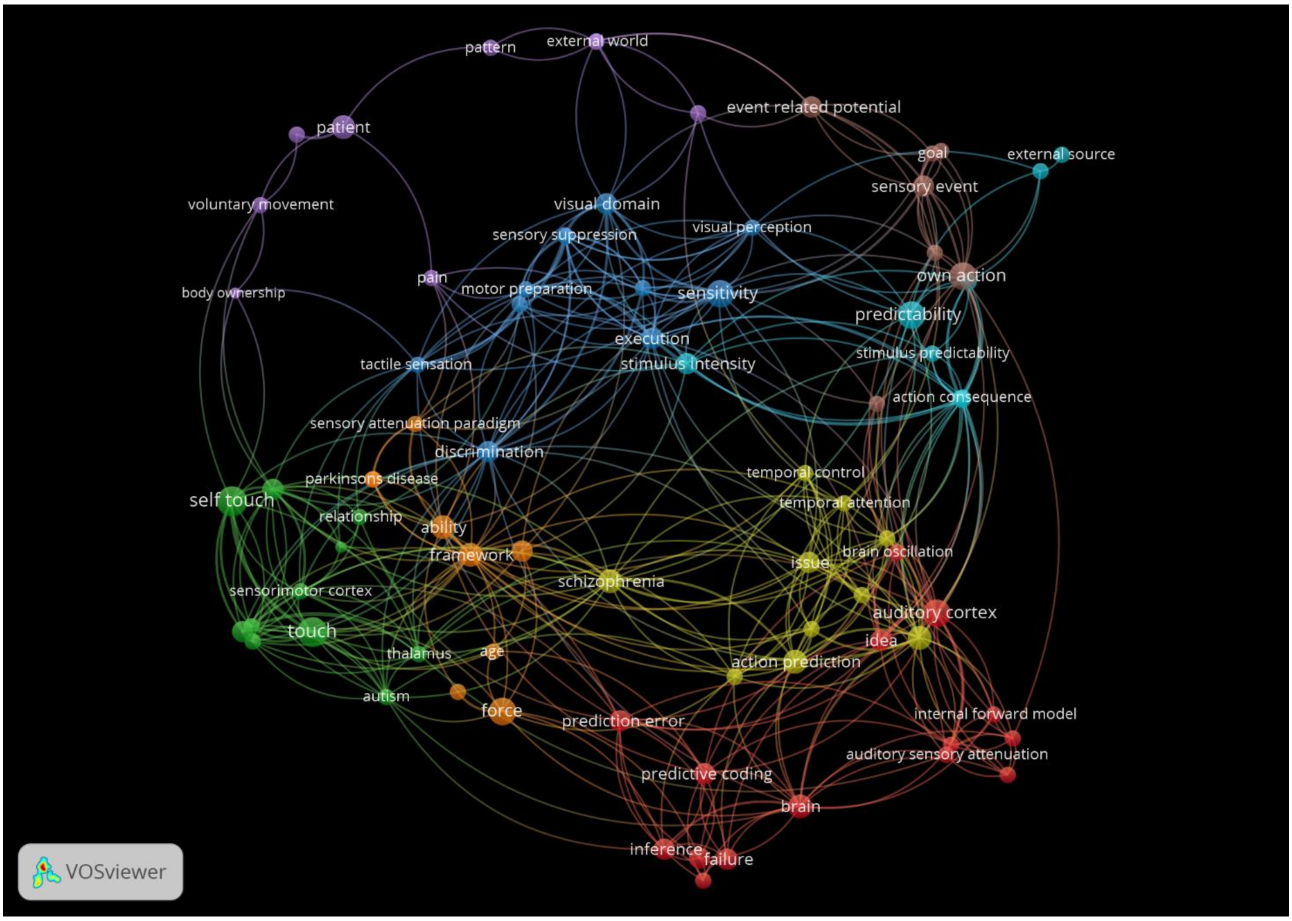


**Figure 4: Network visualization**

- Item density visualization (see Figure 5) & Cluster Density Visualization (see Figure 6):

In Figure 5 the colour of each point (blue, green and yellow) indicates the density of elements at that particular point. In particular, yellow areas identify a point with a higher number and weight of elements in its vicinity. Vice versa for the colour blue. In contrast, in Figure 6, the density of the elements was represented separately for each cluster of elements. The weight given to each colour is determined by the constituent elements of the individual clusters in the areas surrounding the point (van Eck & Waltman, 2023).

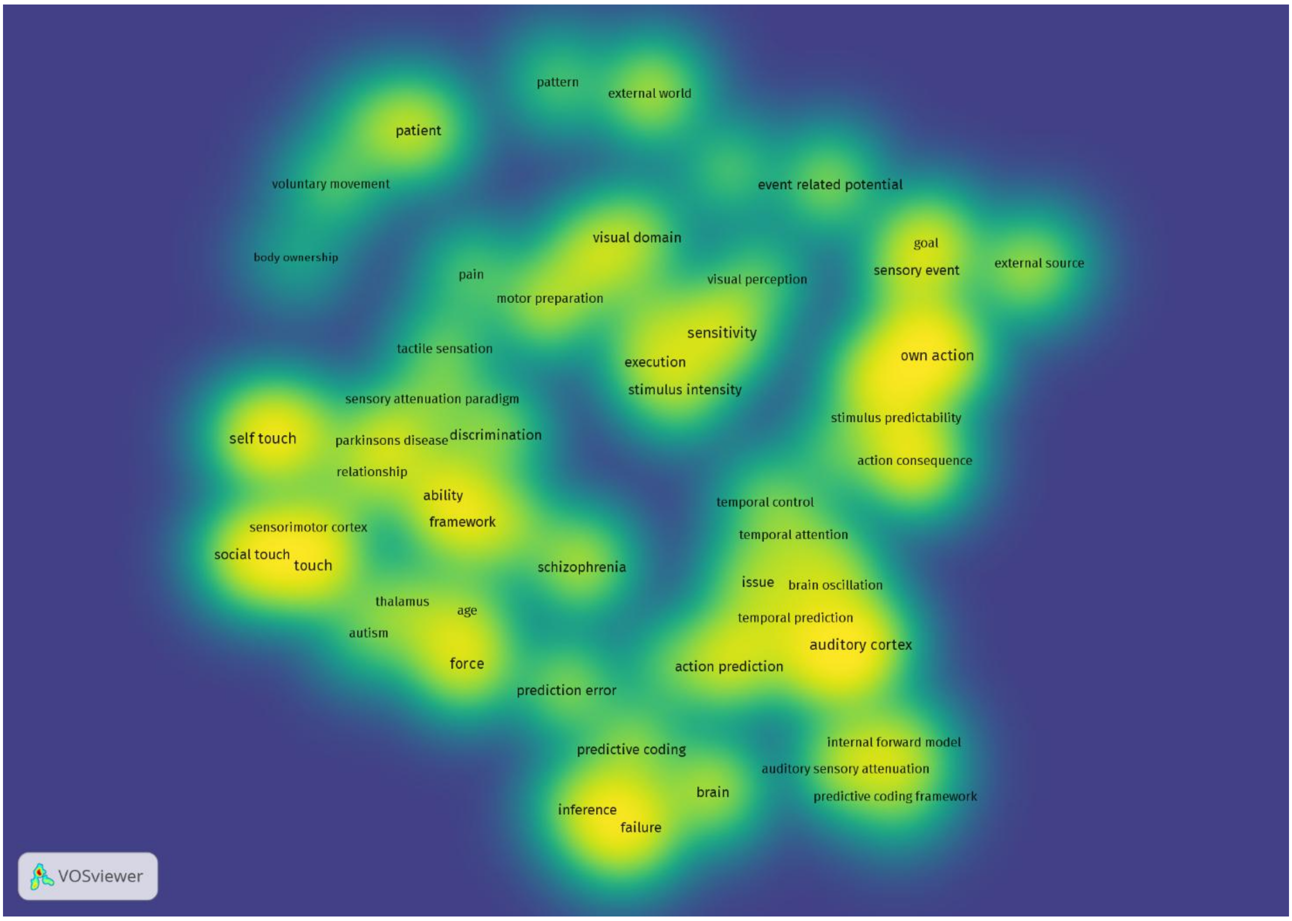


**Figure 5: Item density visualization**

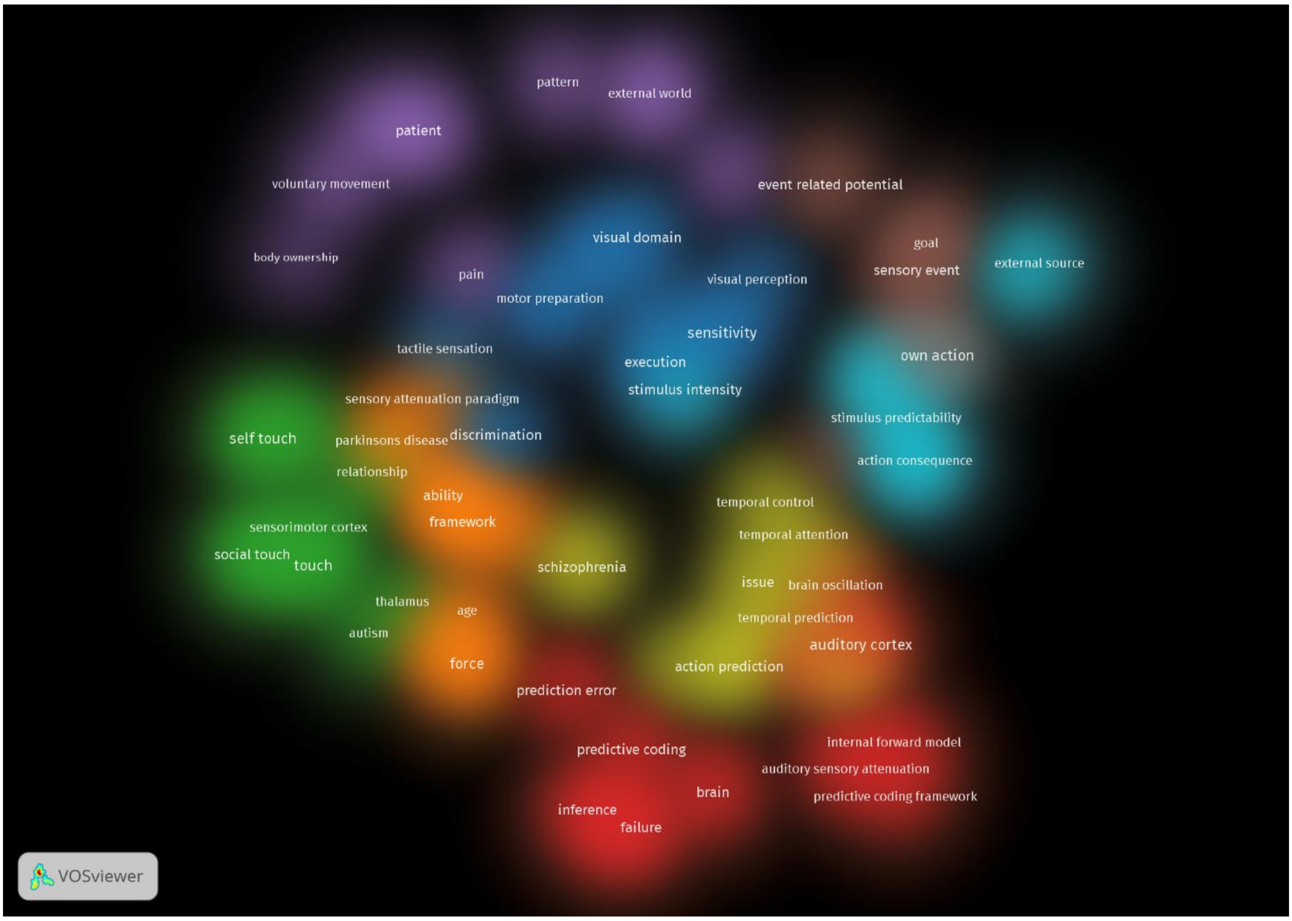


**Figure 6: Cluster density visualization**

References:

van Eck, N.J. and Waltman, L. (2023) ‘VOSviewer’, *VOSviewer - Visualizing scientific landscapes*. Available at: https://www.vosviewer.com/ (Accessed: 2024).
